# Supplementary material for: Heterogeneity of the cancer cell line metabolic landscape
Source: Mol Syst Biol. 2022 Nov 2;18(11):e11006. doi: 10.15252/msb.202211006 (PMC9627668; doi:10.15252/msb.202211006)
Supplement: Supplementary file 1 — Appendix [file MSB-18-e11006-s002.pdf]

Appendix for Shorthouse et al. Heterogeneity of the Cancer Cell Line Metabolic Landscape

Page 2: Appendix Figure S1
Page 4: Appendix Figure S2
Page 5: Appendix Figure S3
Page 7: Appendix Figure S4
Page 8: Appendix Figure S5
Page 9: Appendix Figure S6
Page 11: Appendix Figure S7
Page 12: Appendix Figure S8
Page 13: Appendix Figure S9
Page 14: Appendix Figure S10
Page 16: Appendix Figure S11
Page 17: Appendix Figure S12

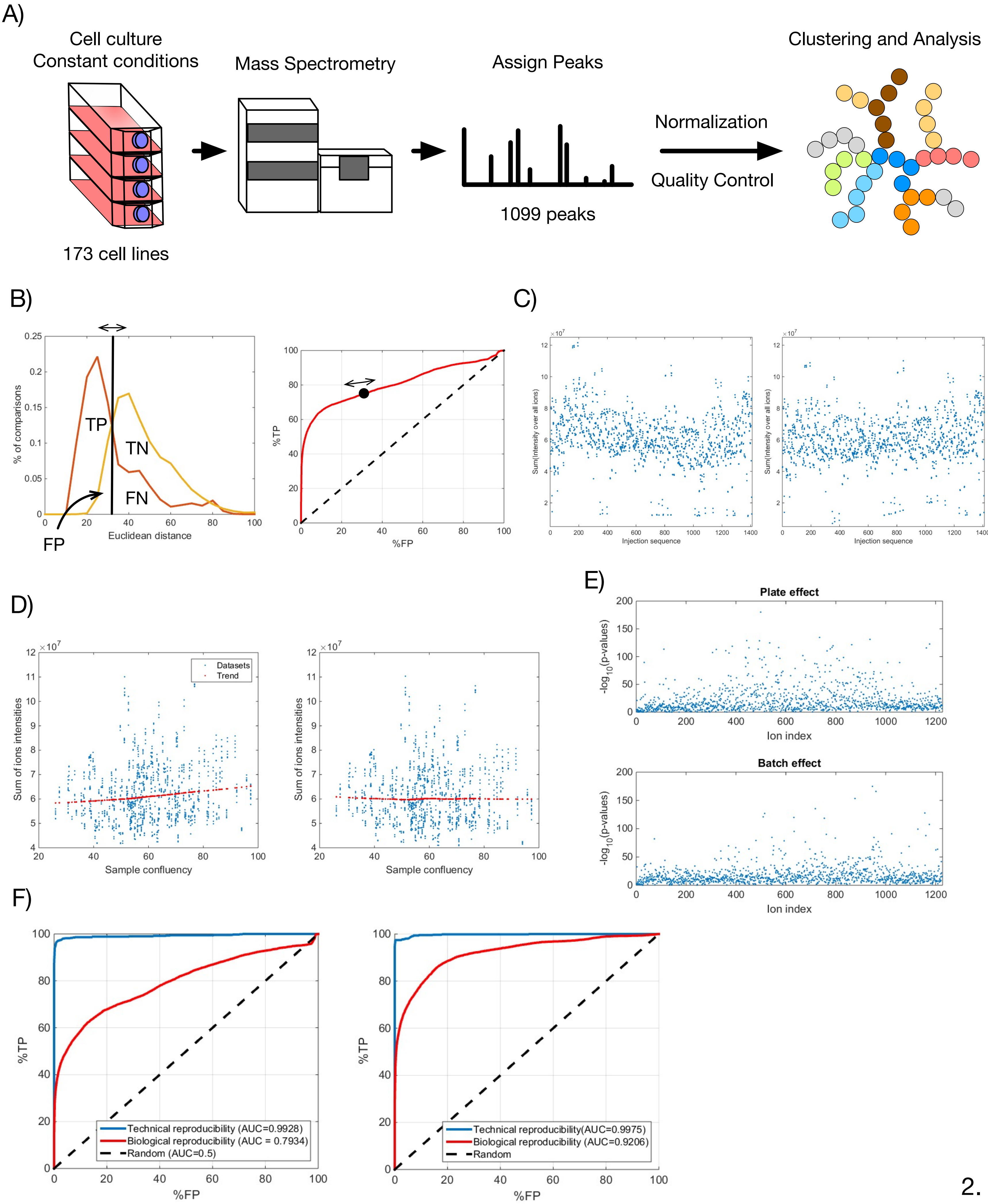
A)
Cell culture
Constant conditions
173 cell lines
Mass Spectrometry
Assign Peaks
1099 peaks
Normalization
Quality Control
Clustering and Analysis
B)
TP
TN
FN
FP
% of comparisons
Euclidean distance
%TP
%FP
C)
Sum(Intensity over all ions)
Injection sequence
D)
Datasets
Trend
Sum of ions intensities
Sample confluency
E)
Plate effect
Batch effect
-log10(p-values)
Ion index
F)
Technical reproducibility (AUC=0.9928)
Biological reproducibility (AUC = 0.7934)
Random (AUC=0.5)
Technical reproducibility(AUC=0.9975)
Biological reproducibility (AUC=0.9206)
Random
%TP
%FP

Appendix Figure S1

A) Schematic of workflow for generating Mass Spectrometry data for 173 cell lines from 11 tissues.

B) Euclidean distance based ROC analysis performed for biological vs non-biological replicates.

C) Total mass per injection before (left) and after (right) Lowess smoothing to control for total injection mass.

D) Total mass per injection before (left) and after (right) Lowess smoothing to control for plate confluence.

E) ANOVA p-values for correlation of each peak with plate (top) and batch (bottom) effects.

F) ROC curves for complete metabolism data before (left) and after (right) normalisation and correction. Red lines represent distances between biological replicates, blue lines represent distances between technical replicates.

A)

B)

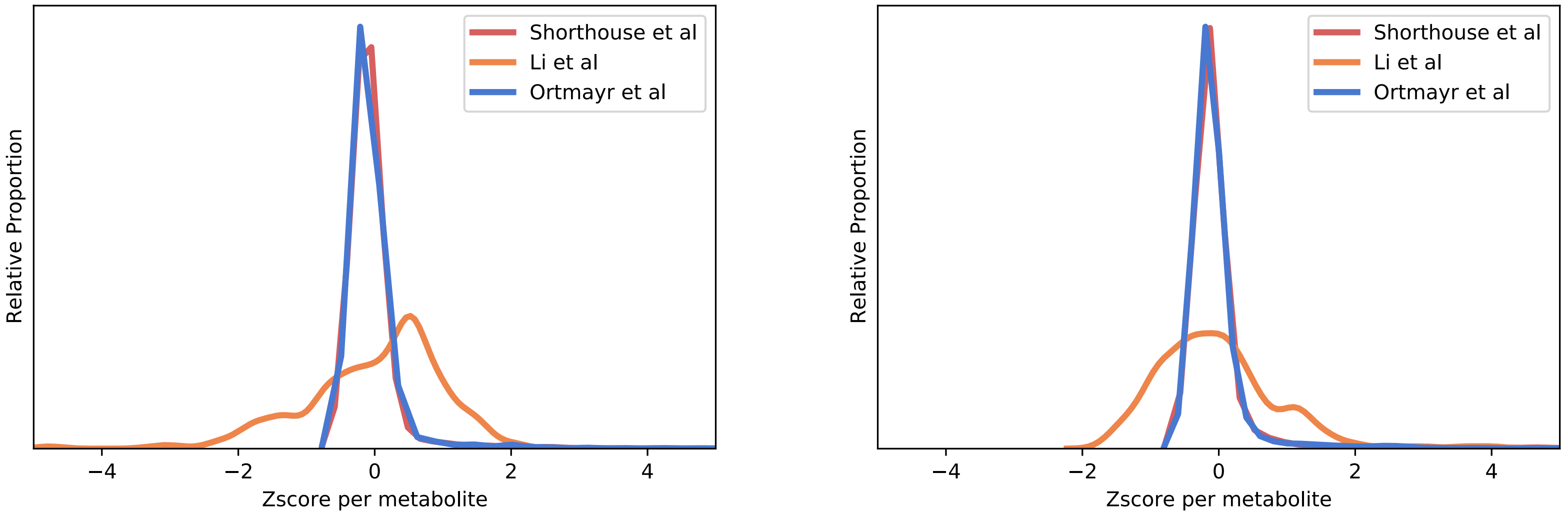

C)

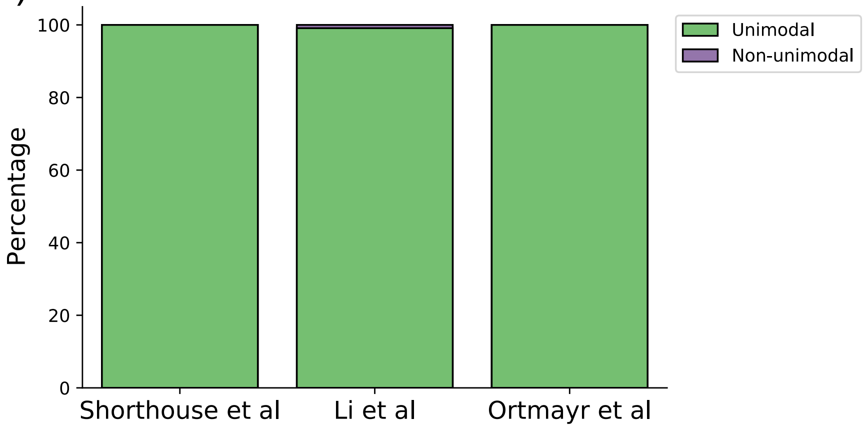

D)

Metabolite	Correlation	Pvalue vs null distribution
2-hydroxglutarate	0.505	0
Glutamine/Glutamic Acid	0.155	0.028
Citrate	0.277	0.0002
Succinate	0.288	0.0002
Glycine	0.16	0.025

Appendix Figure S2

A) Distribution of z-score means for each metabolite in Shorthouse et al, Li et al, and Ortmayr et al

B) Distribution of standard deviations for each metabolite in Shorthouse et al, Li et al, and Ortmayr et al

C) Proportion of unimodal metabolite distributions in Shorthouse et al, Li et al, and Ortmayr et al.

D) Statistical comparison of Shorthouse et al with Li et al. Metabolites from both studies and in overlapping cell lines are correlated using Pearson correlation, and a null distribution generated by permuting one dataset 1,000,000 times. The null distribution is used to generate a pvalue for that metabolite.

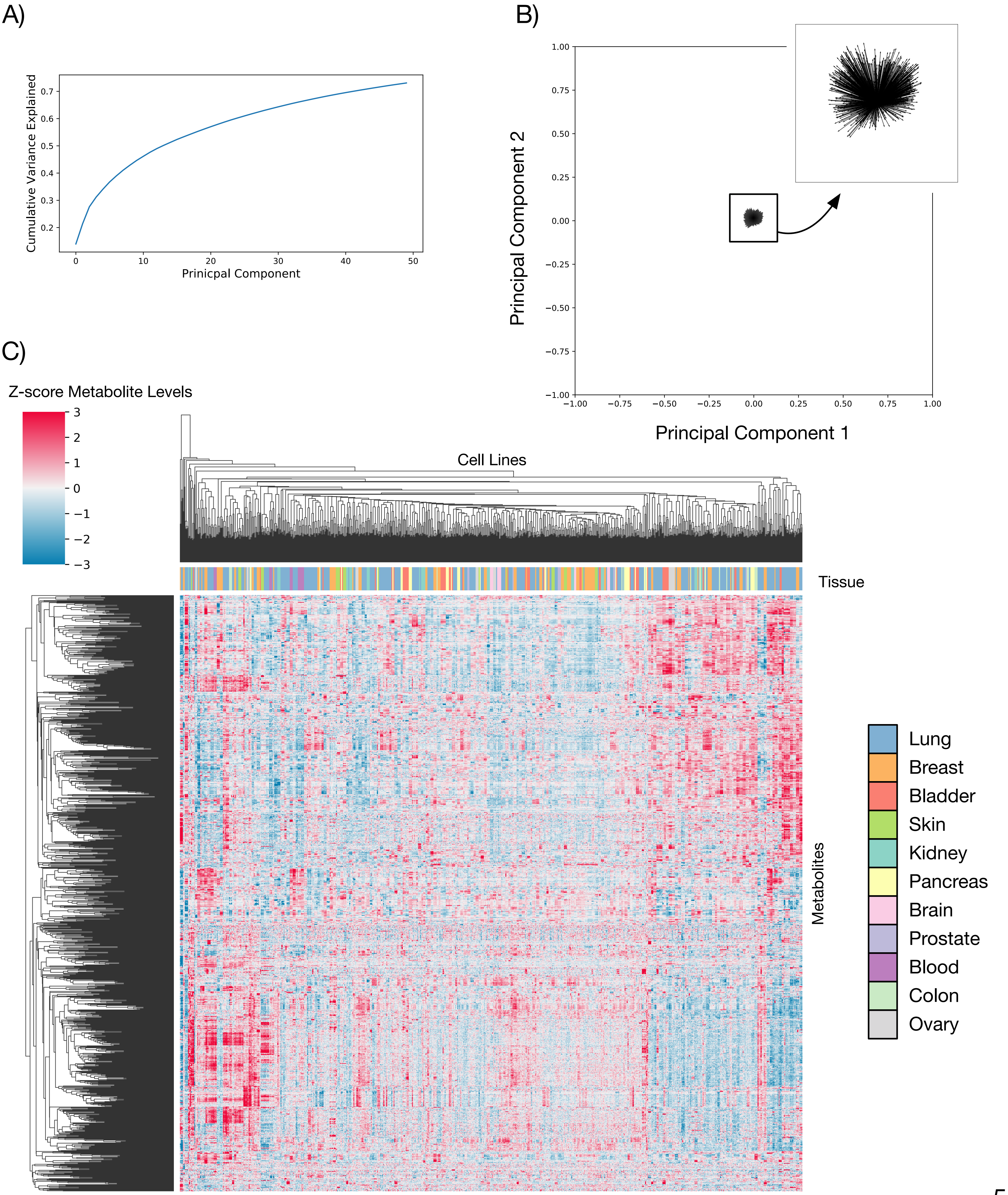
A)
Cumulative Variance Explained
Prinicpal Component
B)
Principal Component 2
Principal Component 1
C)
Z-score Metabolite Levels
Cell Lines
Tissue
Metabolites
Lung
Breast
Bladder
Skin
Kidney
Pancreas
Brain
Prostate
Blood
Colon
Ovary

Appendix Figure S3

A) Cumulative variance explained by principal components of the LC/MS data.
B) Top two principal components of the LC/MS data, each arrow represents the contribution of one peak to the principal component.
C) Heatmap of all metabolite levels in each sample, hierarchically clustered.

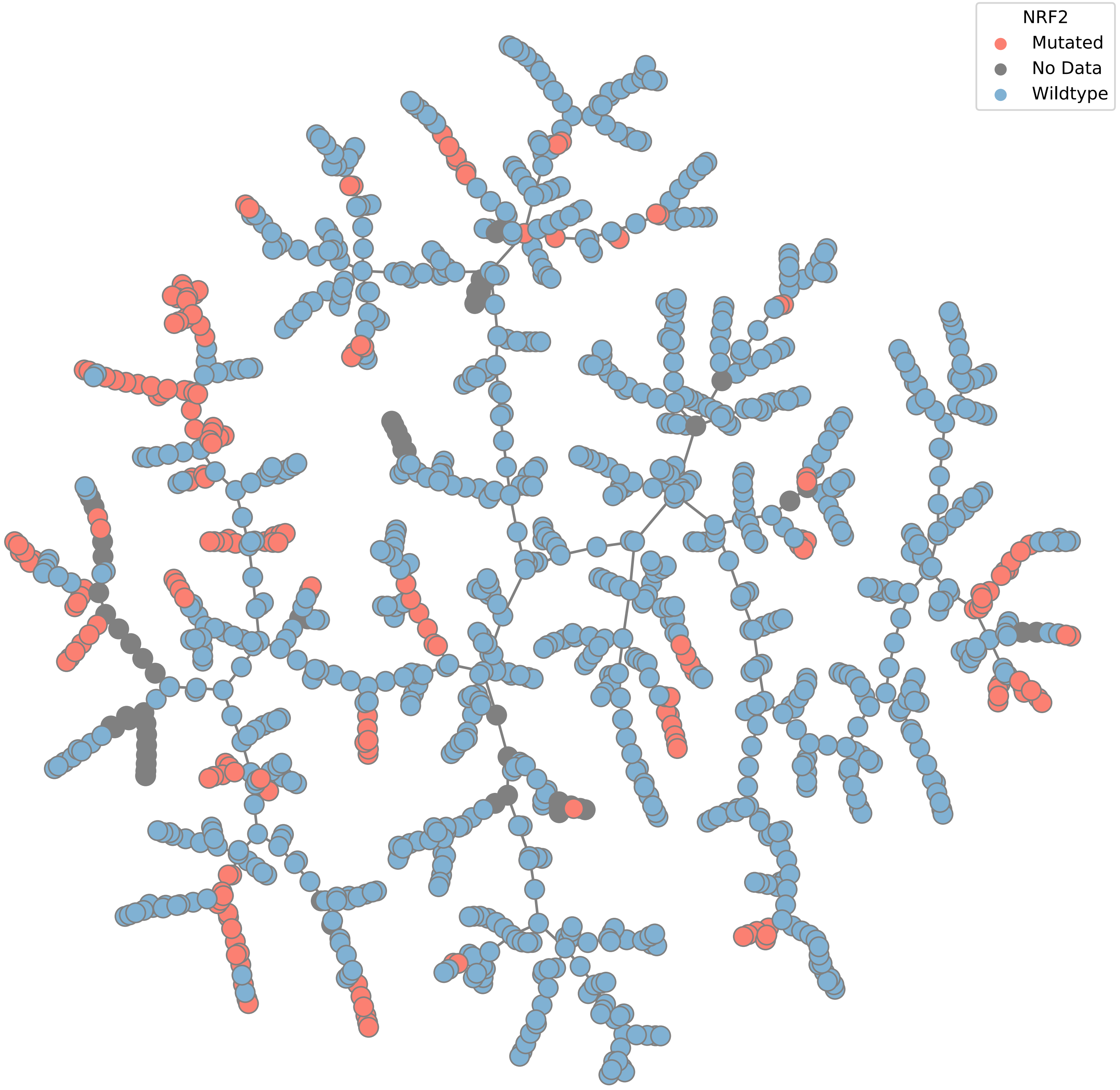

Appendix Figure S4

Tmap for all samples analysed, coloured by presence of a non-silent mutation to any gene in the NRF2 pathway (defined as: NFKB1, NFKB2, RELA, RELB, IKBKB, IKBKE, IKBKG.)

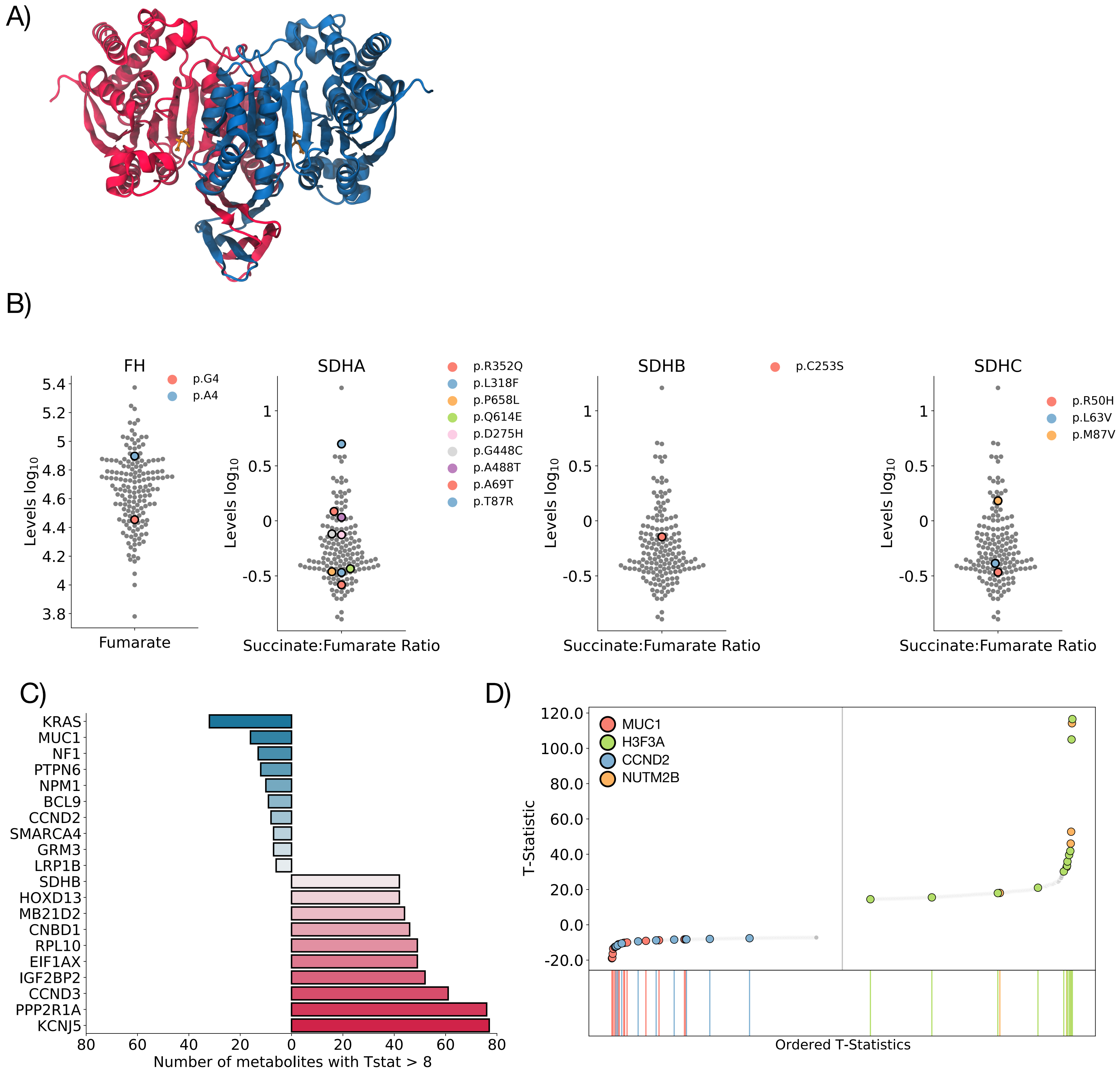

Supplementary Figure 4

Appendix Figure S5

A) Structure of IDH1, colours show two different subunits.

B) Log10 levels for Fumarate (left) or Fumarate:Succinate ratio (others) for FH, SDHA, SDHB, and SDHC. Coloured dots represent samples with a non-silent mutation in the gene.

C) Top and bottom 10 genes ranked according to the number of metabolites with an absolute T-statistic above 8.

D) Rank plot of the T statistics for mutations in MUC1, H3F3A, CCND2, and NUT2MB.

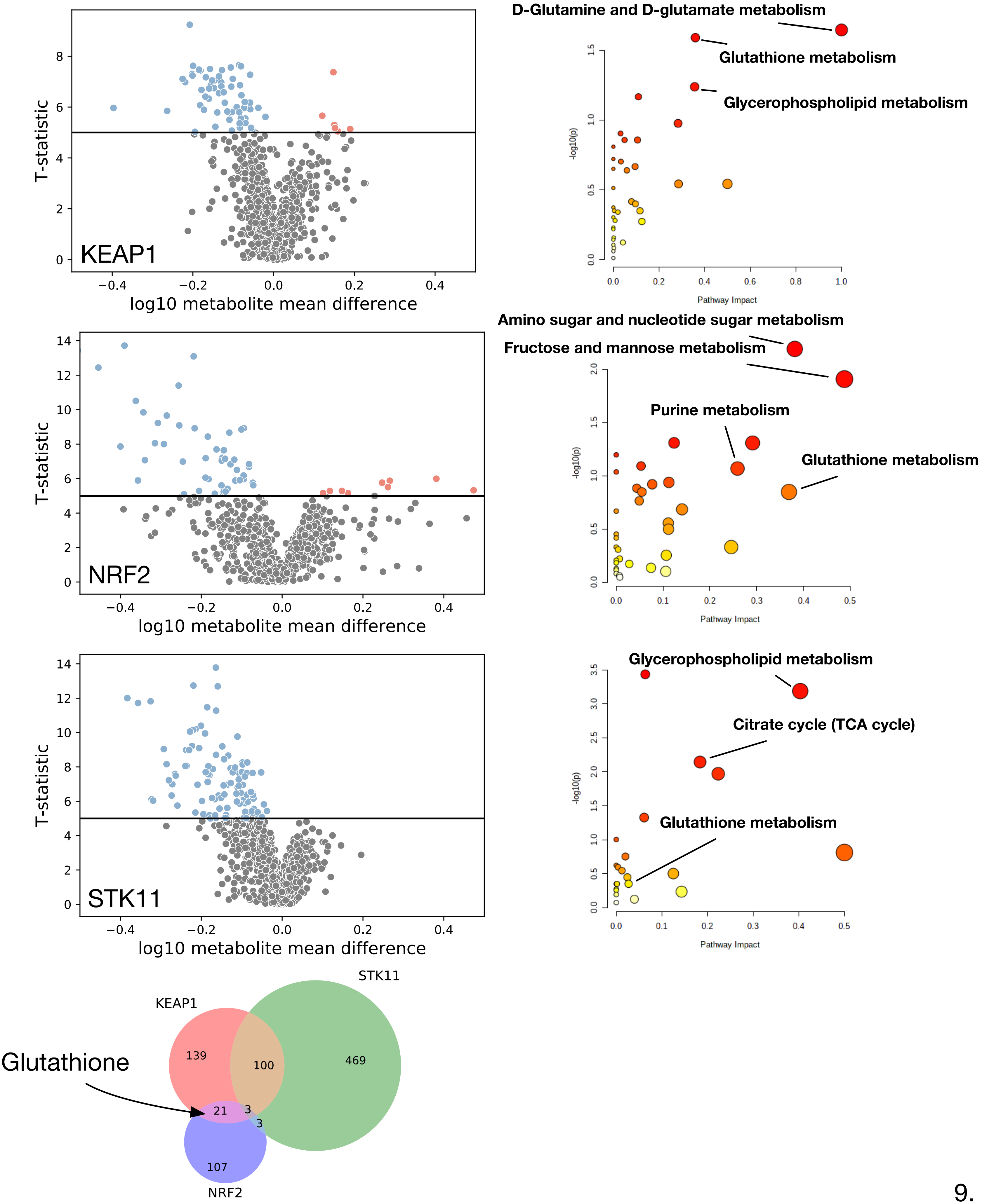
KEAP1
NRF2
STK11
T-statistic
log10 metabolite mean difference
D-Glutamine and D-glutamate metabolism
Glutathione metabolism
Glycerophospholipid metabolism
Amino sugar and nucleotide sugar metabolism
Fructose and mannose metabolism
Purine metabolism
Glutathione metabolism
Glycerophospholipid metabolism
Citrate cycle (TCA cycle)
Glutathione metabolism
-log10(p)
Pathway Impact
Glutathione
KEAP1
STK11
NRF2
139
100
469
21
3
3
107

Appendix Figure S6

Differently expressed metabolites for lung cell lines mutated for KEAP1 (top), NRF2 (middle), and STK11 (bottom). Shown are volcano plots of log10 metabolite mean difference between mutated and wildtype cell lines against regression T statistic (left), and pathway enrichment of all metabolites with a T-statistic > 5 (right). Bottom shows a Venn diagram of the overlap between significant metabolites (T-statistic >5) in all 3 mutant cases.

A)

KEGG Pathway	Raw pvalue	FDR	Impact
Aminoacyl-tRNA biosynthesis	5.74E-05	0.004825	0.16667
Pantothenate and CoA biosynthesis	0.0010016	0.042067	0.46786
Phenylalanine, tyrosine and tryptophan biosynthesis	0.0029319	0.078127	1
Valine, leucine and isoleucine biosynthesis	0.0037203	0.078127	0
Glycine, serine and threonine metabolism	0.0084966	0.11641	0.63031
Phenylalanine metabolism	0.0096216	0.11641	0.61904
Sphingolipid metabolism	0.0097009	0.11641	0.44828
D-Glutamine and D-glutamate metabolism	0.012736	0.13373	0
beta-Alanine metabolism	0.038713	0.36132	0.61567
Histidine metabolism	0.053737	0.45139	0.54917

B)

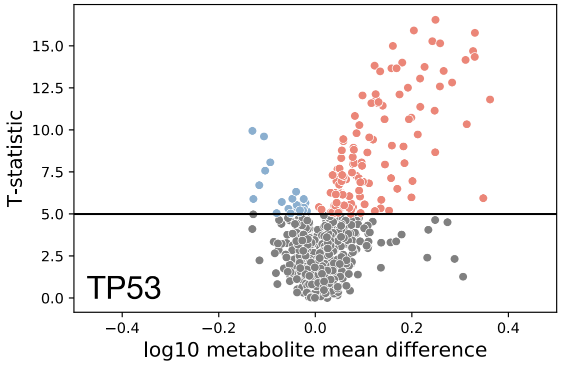

KEGG Pathway	Raw p	FDR	Impact
Pentose and glucuronate interconversions	0.023139	0.54688	0.26562
Amino sugar and nucleotide sugar metabolism	0.025709	0.54688	0.38174
Alanine, aspartate and glutamate metabolism	0.027995	0.54688	0.10817
Fructose and mannose metabolism	0.033248	0.54688	0.48792
Inositol phosphate metabolism	0.036732	0.54688	0.12365
Sphingolipid metabolism	0.039132	0.54688	0.20284
Pentose phosphate pathway	0.045574	0.54688	0.11117
Glycerophospholipid metabolism	0.072135	0.64571	0.413
Glycerolipid metabolism	0.075296	0.64571	0.10592
Glycolysis / Gluconeogenesis	0.076871	0.64571	0.17541

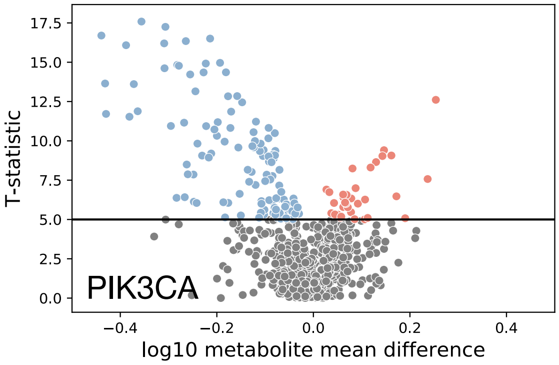

KEGG Pathway	Raw p	FDR	Impact
Alanine, aspartate and glutamate metabolism	0.00027622	0.023202	0.44791
Arginine biosynthesis	0.0013909	0.058416	0.11675
Glycine, serine and threonine metabolism	0.0044787	0.1254	0.20116
Valine, leucine and isoleucine biosynthesis	0.012156	0.25527	0
Aminoacyl-tRNA biosynthesis	0.034151	0.57373	0
Pyruvate metabolism	0.05163	0.64856	0.0622
D-Glutamine and D-glutamate metabolism	0.054046	0.64856	0
Glycerophospholipid metabolism	0.082741	0.80642	0.25152
Glycolysis / Gluconeogenesis	0.086403	0.80642	0.31271
Pantothenate and CoA biosynthesis	0.12455	0.9037	0.02143

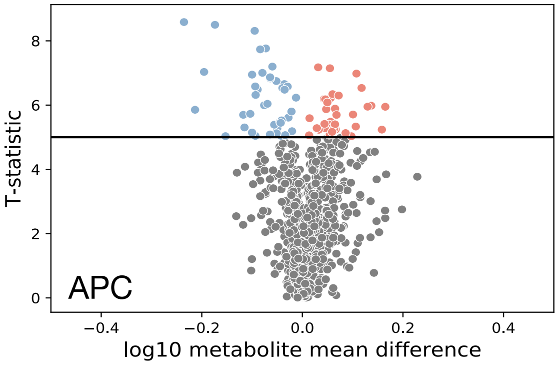

KEGG Pathway	Raw p	FDR	Impact
Amino sugar and nucleotide sugar metabolism	6.57E-09	5.52E-07	0.54489
Galactose metabolism	2.93E-05	0.0012316	0.1605
Fructose and mannose metabolism	0.0024258	0.063352	0.48792
Inositol phosphate metabolism	0.0030167	0.063352	0.25304
Ascorbate and aldarate metabolism	0.0057192	0.096083	0.5
Glycolysis / Gluconeogenesis	0.0081132	0.11123	0.07791
Pentose and glucuronate interconversions	0.010593	0.11123	0.20312
Starch and sucrose metabolism	0.010593	0.11123	0.30137
Phosphatidylinositol signaling system	0.048428	0.452	0.09215
Neomycin, kanamycin and gentamicin biosynthesis	0.098144	0.82441	0

Appendix Figure S7

A) Pathway enrichment table for metabolites significantly associated with KRAS mutations (T-statistic > 5)

B) Volcano plots and pathway enrichment tables for metabolites significantly associated (T-statistic >5) with TP53 (left), APC (middle), and PIK3CA (right).

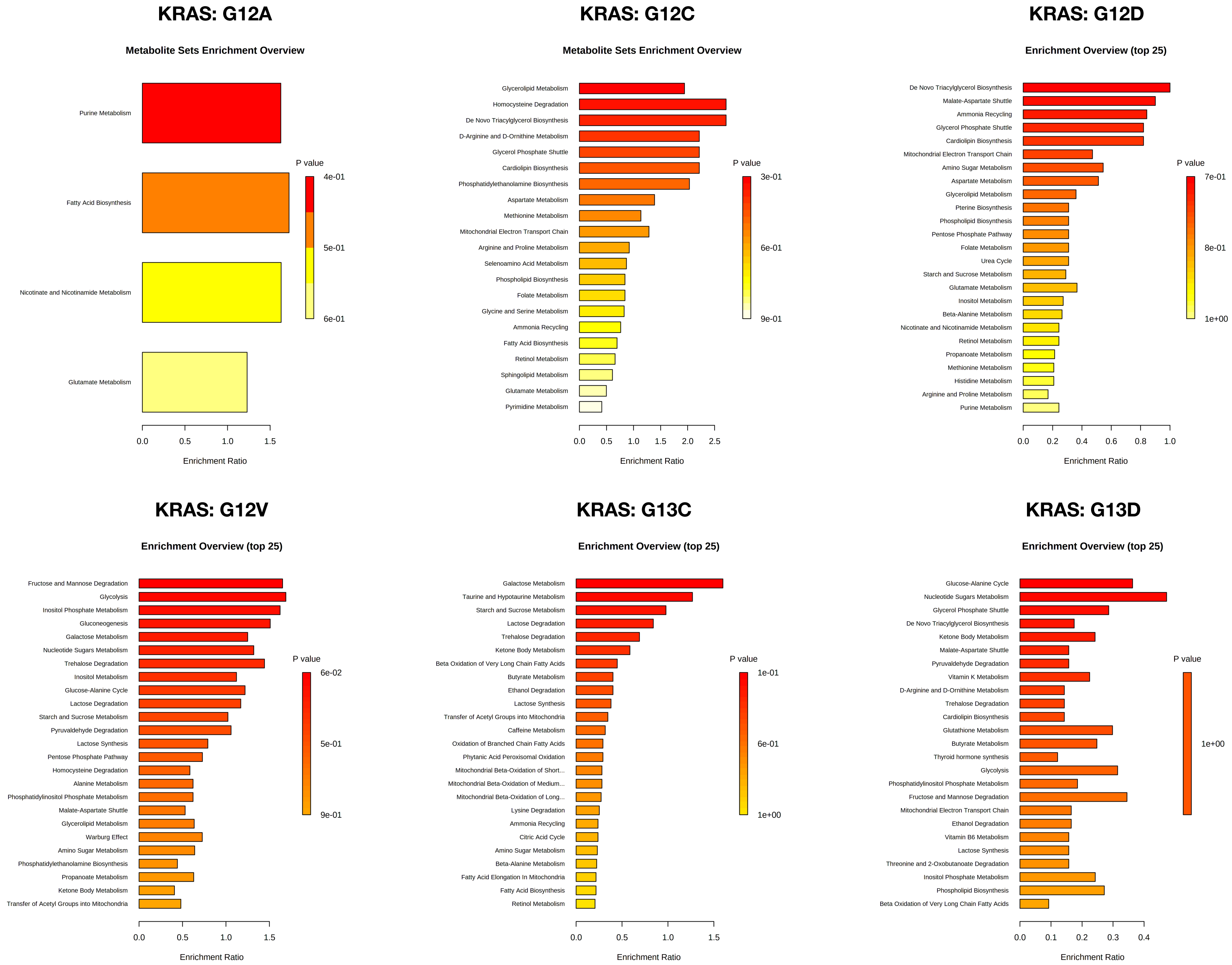

Appendix Figure S8

Enriched metabolic pathways for metabolites with a Tstat greater or lower than 5 for different KRAS mutations.

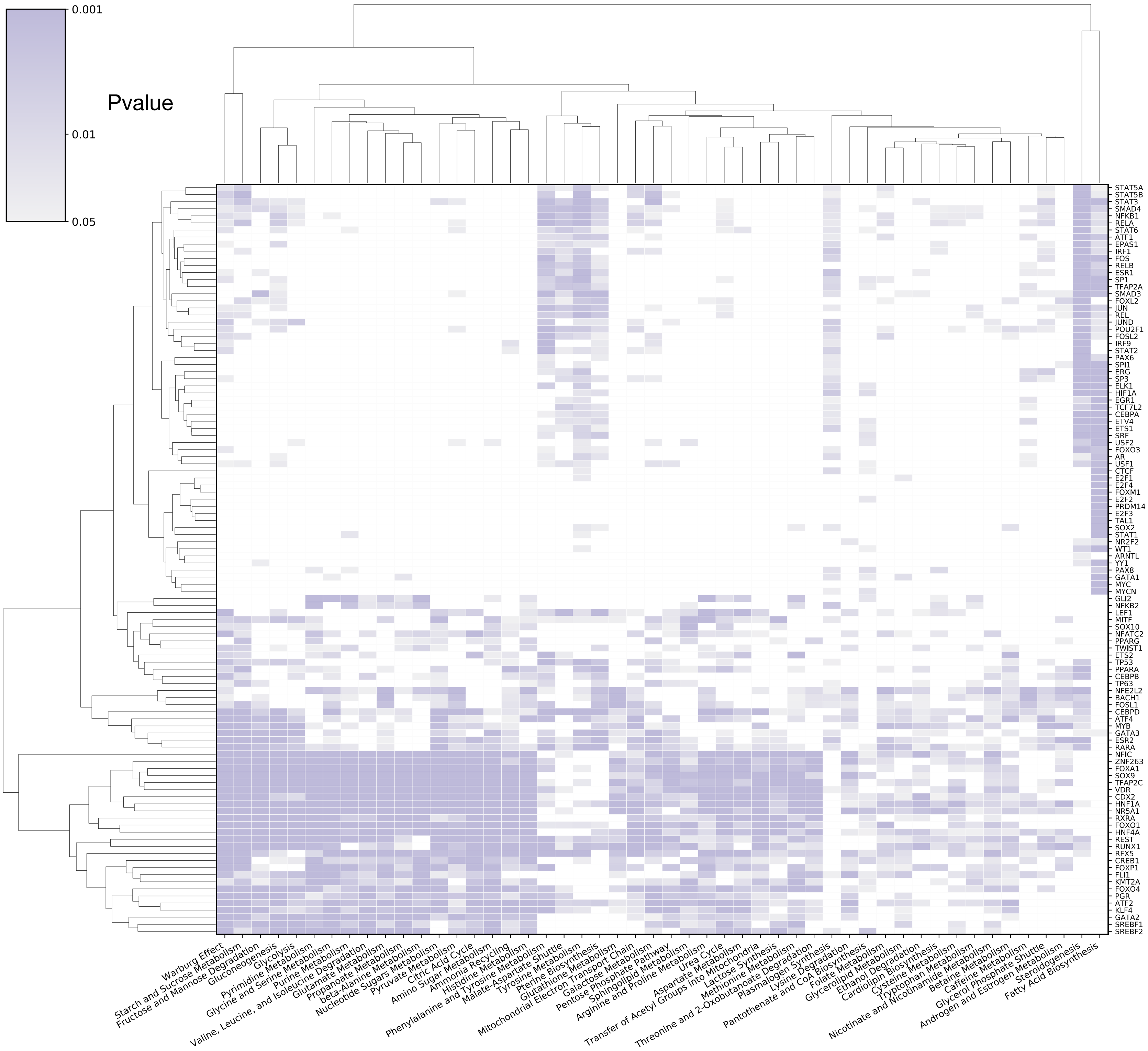

Appendix Figure S9

Heatmap of p-values (hypergeometric test) between SMPDB core metabolic pathways (x axis) and DOROTHEA (A+B significant) calculated transcriptional regulon activity (y axis)

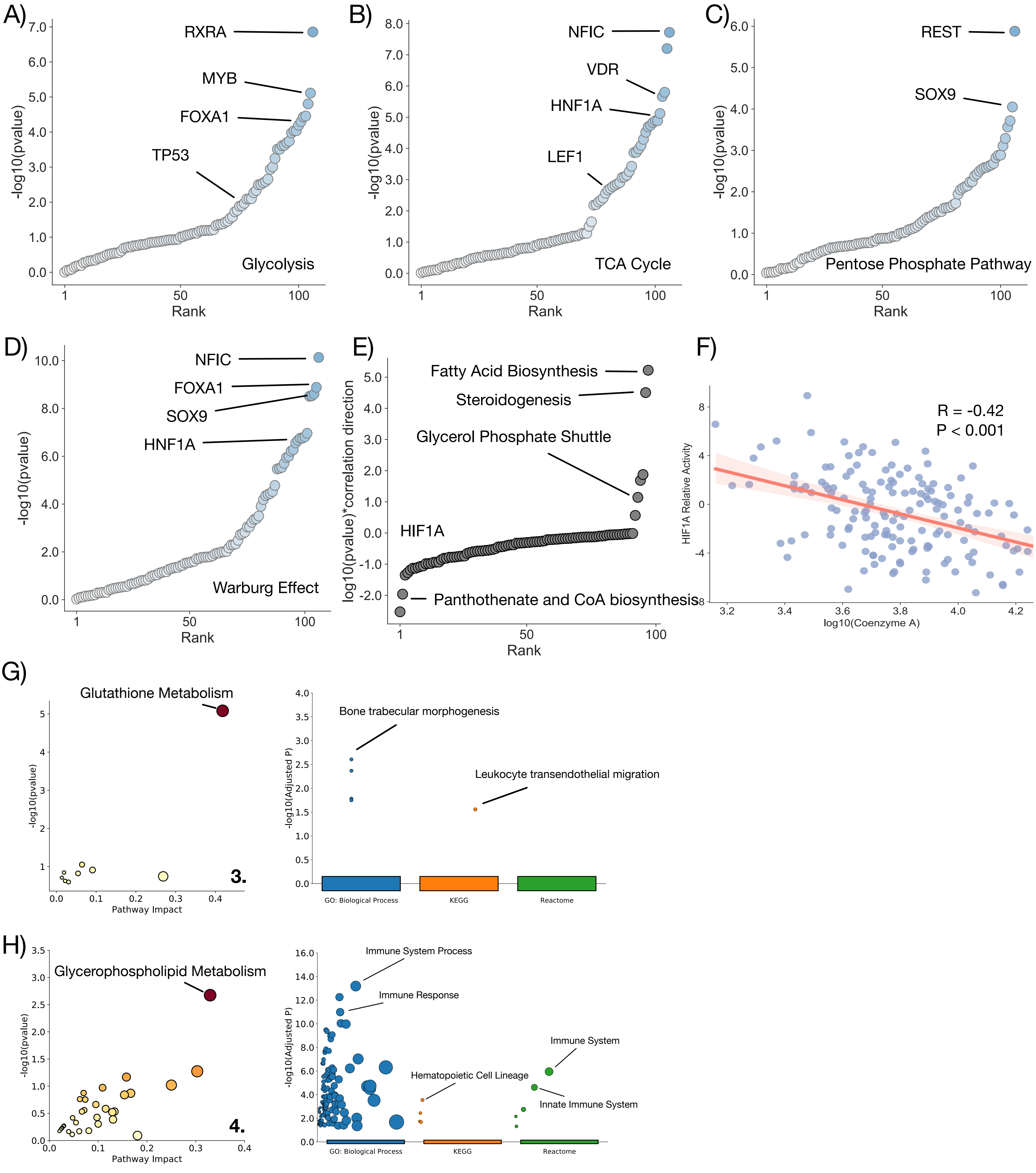
A)
RXRA
MYB
FOXA1
TP53
-log10(pvalue)
Rank
Glycolysis
B)
NFIC
VDR
HNF1A
LEF1
-log10(pvalue)
Rank
TCA Cycle
C)
REST
SOX9
-log10(pvalue)
Rank
Pentose Phosphate Pathway
D)
NFIC
FOXA1
SOX9
HNF1A
-log10(pvalue)
Rank
Warburg Effect
E)
Fatty Acid Biosynthesis
Steroidogenesis
Glycerol Phosphate Shuttle
HIF1A
Panthothenate and CoA biosynthesis
log10(pvalue)*correlation direction
Rank
F)
R = -0.42
P < 0.001
HIF1A Relative Activity
log10(Coenzyme A)
G)
Glutathione Metabolism
-log10(pvalue)
Pathway Impact
3.
Bone trabecular morphogenesis
Leukocyte transendothelial migration
-log10(Adjusted P)
GO: Biological Process
KEGG
Reactome
H)
Glycerophospholipid Metabolism
-log10(pvalue)
Pathway Impact
4.
Immune System Process
Immune Response
Immune System
Hematopoietic Cell Lineage
Innate Immune System
-log10(Adjusted P)
GO: Biological Process
KEGG
Reactome

Appendix Figure S10

A) -log10 pvalue rank plot for all DOROTHEA A+B significant transcriptional regulons and their correlation against the Glycolysis SMPDB pathway.
B) -log10 pvalue rank plot for all DOROTHEA A+B significant transcriptional regulons and their correlation against the TCA Cycle SMPDB pathway.
C) -log10 pvalue rank plot for all DOROTHEA A+B significant transcriptional regulons and their correlation against the Pentose Phosphate SMPDB pathway.
D) -log10 pvalue rank plot for all DOROTHEA A+B significant transcriptional regulons and their correlation against the Warburg Effect SMPDB pathway.
E) -log10 pvalue * correlation direction plot for all SMPDB pathways against the HIF1A activity score.
F) log10(Coenzyme A) levels against HIF1A relative activity.
G) Metabolic pathway enrichment (left) and genetic pathway enrichment (right) for group 3 in Figure 3D.
H) Metabolic pathway enrichment (left) and genetic pathway enrichment (right) for group 4 in Figure 3D.

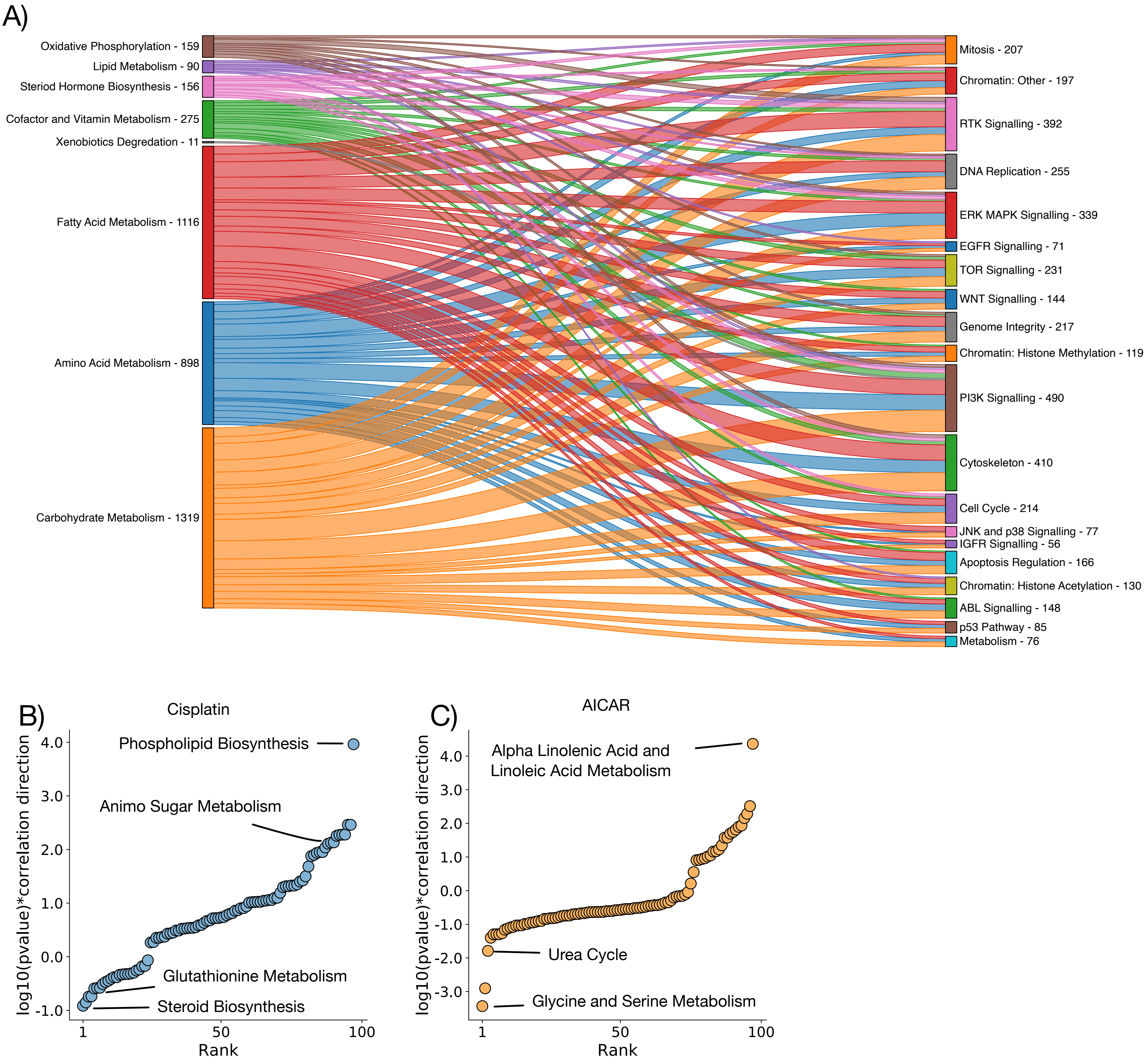

Appendix Figure S11

A) Sankey plot for significant ($p < 0.05$) associations between classes of metabolic pathways (left), and drug activities (right)

B) log10 pvalue * correlation direction for correlation between SMPDB pathways and Cisplatin.

C) log10 pvalue * correlation direction for correlation between SMPDB pathways and AICAR.

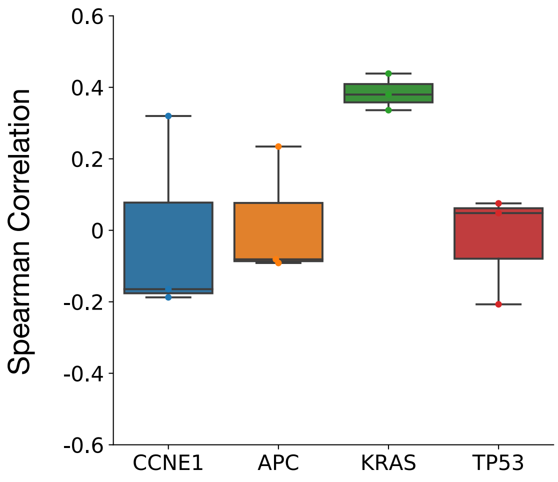

Appendix Figure S12

Boxplot of the spearman correlations between pairs of metabolite differences between mutant and WT samples for Bladder, Breast, and Lung cancer.
