# Supplementary material for: Heterogeneity of the cancer cell line metabolic landscape
Source: Mol Syst Biol. 2022 Nov 2;18(11):e11006. doi: 10.15252/msb.202211006 (PMC9627668; doi:10.15252/msb.202211006)
Supplement: Supplementary file 11 [file MSB-18-e11006-s008.pdf]

molecular systems biology

Heterogeneity of the cancer cell line metabolic landscape

David Shorthouse, Jenna Bradley, Susan Critchlow, Claus Bendtsen, and Benjamin Hall
DOI: 10.15252/msb.202211006

Corresponding author(s): Benjamin Hall (b.hall@ucl.ac.uk)

Review Timeline:

Submission Date:	17th Mar 22
Editorial Decision:	22nd Apr 22
Revision Received:	4th Aug 22
Editorial Decision:	30th Aug 22
Revision Received:	30th Aug 22
Accepted:	7th Oct 22

Editor: Jingyi Hou

Transaction Report:

(Note: With the exception of the correction of typographical or spelling errors that could be a source of ambiguity, letters and reports are not edited. Depending on transfer agreements, referee reports obtained elsewhere may or may not be included in this compilation. Referee reports are anonymous unless the Referee chooses to sign their reports.)

1st Editorial Decision — 22nd Apr 2022

Thank you for submitting your work to Molecular Systems Biology. We have now heard back from the three reviewers who agreed to evaluate your manuscript. As you will see from the reports below, the reviewers acknowledge the relevance and potential interest of the study. They raise, however, a series of concerns, which we would ask you to address in a major revision.

I think the reviewers' recommendations are relatively straightforward, and there is no need to reiterate their comments. In particular, some of the key issues that would need to be addressed are the following:

- A direct comparison with the existing metabolomic data.
- Addressing the concerns about statistics, pathway score analysis, and several potential confounding factors in the association analysis.
- In line with Reviewers #1 and #2's comments, please improve the clarity regarding the origin and processing procedure of the metabolomics data. The data processing differences between this study and Cherkaoui et al study(if any) should be clearly stated in the Materials and Methods section.
- In light of Reviewer #2's suggestion, we would strongly encourage the provision of an online tool to enhance the usability and accessibility of the data.

Other issues raised by the reviewers need to be satisfactorily addressed as well. As you may already know, our editorial policy allows, in principle, a single round of major revision, and acceptance or rejection of the manuscript will depend on another round of review, it is therefore essential to provide responses to the reviewers' comments that are as complete as possible.

On a more editorial level, we would ask you to address the following issues:

Reviewer #1:

In the present manuscript, Shorthouse et al investigate the landscape of metabolic profiles of 173 cancer cell lines grown under identical conditions. Using metabolomics data generated as part of an accompanying back-to-back manuscript (Cherkaoui et al) as their starting point, the authors integrate various types of orthogonal information to identify e.g. associations between metabolites and gene mutations, or between metabolic pathway activity and drug sensitivity.
Overall, this manuscript is well written and contains a plethora of interesting vignettes that will surely be of interest for the readership of MSB. However, there are currently several issues that should be clarified in a revised manuscript.

Main comments:
1. Origin of metabolomics data: it is my understanding that the metabolomics data used by the authors is actually the same as the one generated by the accompanying manuscript from the Zamboni lab. However, this point is not actually spelled out in the text, and the methods section gives the somewhat misleading impression that this work also generated metabolomics measurements. I strongly suggest that the authors of both manuscripts clarify this point in their revised manuscript.

2. Processing of metabolomics data: More importantly, it is unclear to me why this work used a quite different processing/normalization pipeline compared to the one from Cherkaoui et al, in which not only the number of ions considered, but also the number of cell lines are different. After doing some spot checks, I realized that the intensities for the same ions in the same samples seem to be quite different in the two studies (comparing suppl table 1 of this manuscript and the "injections" sheet of the Metabolomics data set provided as Primary analysis in the Zamboni lab manuscript), suggesting substantial differences in the output of the normalization pipelines. Given that the underlying data are the same (and taken from the Zamboni lab manuscript), I do not understand why the authors used a different normalization pipeline. If there is indeed a good reason for this decision, it is currently not communicated in the manuscript. I strongly suggest that the authors (of both manuscripts) either harmonize their data normalization pipelines or explain in detail why different ones are being used. Otherwise, I worry that having two conflicting data sets based on the same original data published back-to-back will lead to substantial confusion in the field.

3. Relationship to previous literature: as the authors point out themselves, this is not the first attempt to comprehensively access the metabolic state of diverse cancer cell lines. For example, recent works have covered a larger cell panel, but with fewer metabolites measured (PMID 31068703), or a smaller cell panel (~60 cell lines), but with a similar number of measured metabolites (PMID 31015463). However, in the present manuscript the authors make little effort to put their findings in the broader context of the recent literature. For example, do any of the high-level conclusions of these recent studies hold up (or change) when expanding the metabolite/cell line dimension? I believe that this manuscript would benefit from an additional paragraph e.g. in the discussion tackling this point. Especially since it will allow the authors to articulate more clearly the "added value" this study provides to the field (which currently remains somewhat unclear).

4. Figure 4B: I got confused regarding the exact metric being used here. In my understanding, the authors correlate (for a given drug) the IC50 value with a metabolic pathway activity metric across cell lines. A negative correlation between these two metrics would mean that higher pathway activity is associated with lower IC50 (and thus higher sensitivity to the respective drug). Since the log10 of the p-value is always going to be negative, I therefore expected positive products of log10(p-val) * Correlation_Direction to signify sensitivity. However, in figure 4B these are labeled as "resistant". Did the authors miss a (-) sign before the log10(p-val), or am I missing something?

5. In general, I found Figures 4B-D very intriguing but hard to digest. For example, figure 4C is very interesting, but it didn't become clear to me a) why these particular two metabolic pathways were being highlighted here, and b) what the coloring scheme signifies. Similarly, in Figure 4D, I wasn't sure what type of correlation is being calculated here: is it the correlation of metabolic pathway sensitivities between different drugs? I suggest that authors add more detail on what exactly is being calculated in these plots.

Additional comments:
1. Terminology: throughout the text, the authors refer to "upregulated" or "differently expressed" metabolites, and in one instance mention a mutation that is associated with a "loss of glutamine" (lines 249-250). I understand what the authors are trying to say, but I suggest that these terms (which make sense for gene expression, but not for metabolites) should be changed to e.g. "increased", "differently abundant". Similarly, I would suggest that the authors change the term "smaller doubling time" to the more conventional "shorter doubling time".

2. Figure S1: in my understanding, the metabolomics method uses direct injection, without any chromatographic separation. To avoid confusion, I suggest that the authors remove the "LC-" prefix from panel A.

3. Line 140: I assume it's supposed to be Figure S2B (and not 2B), right? I also wasn't sure how to interpret this figure (and the

accompanying lines 138-140): the argument is that all arrows in this plot have similar lengths, suggesting that no individual peak has a dominating effect on the PC's? An additional half-sentence may help clarify this point.

4. Clustering shown in Figure 1A: The authors conclude that cell lines from the same tissue are generally clustering together (lines 147-148). I found this hard to see, especially since almost half of the cell lines are from the lung. Is there a way to provide a statistical measure of the "homogeneousness" of the different branches to back up this conclusion?

5. The authors' efforts to find associations between metabolites and gene mutations is very intriguing, in particular the associations between metabolic enzymes and their substrates/products. The IDH1 example the authors provide is illuminating (suggesting that only one of the two mutations found in the cell lines affects IDH1 catalytic activity). Have the authors considered to systematically look for such enzyme-substrate/product pairs to identify those mutations that may affect catalytic activity?

6. Figure 5B: the authors refer to NRAS and BRAF in the text, but I couldn't see the respective data in Figure 5B (only KRAS and HRAS).

Reviewer #2:

The paper from Shorthouse et al. presents a potentially incredibly useful data resource. They generated untargeted metabolomic profiling data entailing 1099 ions for 173 cancer cell lines. The authors performed extensive association analysis with mutation, transcriptomic, and drug sensitivity data, with most of the analyses performed at the pathway level. However, most of the analyses seem to be quite superficial, without in-depth functional validation.

Major points:
1. Throughout the paper, the author kept replicate data in their analyses. I highly recommend they collapse the data to cell line-level, i.e. pick only one representative "centroid" cell line and remove the other replicates.
2. Inter-replicate variation is much smaller than inter-cell strain variation. None of the statistical tests they performed has taken into account these different levels of variation.
3. The data representation by including replicates might also be misleading. For example in 1A, the local clusters of replicates from the same cell line make it seem like cell lines of the same tissue origin cluster tighter than they really are.
4. In Figure 2, the authors tried to state that different mutations in IDH1 or SDHAF22 can result in different changes in the downstream metabolite. Such claims require validation by replacing WT IDH1 or SDHAF2 in the same reference cell line with the point mutations of interest to see if the metabolic changes from the parental cell line match with what they observed in Figures 2A and 2C. In the current analysis, the 6 dots for each mutation are really from 6 replicates of the same cell line. These different point mutations were compared in different cell line backgrounds, not isogenic backgrounds. They need to point out the cell lines for each of the different IDH1 or SDHAF2 mutations.
5. Pathway score was calculated and analyzed extensively in the paper. However, with shotgun metabolomics, the same ion could match many different metabolites. If these isobaric metabolites happen to fall into the same pathway, wouldn't the pathway score be inflated as an artifact? Can the authors show how would the results be affected if only one most abundant representative metabolite is picked for the ion?
6. I would like to see a comparison (distribution of metabolite-specific pairwise correlations across shared cell lines for all the shared metabolites) with the existing CCLE metabolomics data to see the cross-study reproducibility of cell line metabolomics data.
7. Have p-values been adjusted for multiple comparisons for all of the related analyses (I only see it for RNA correlation)? If not, they should. Please indicate in the text accordingly.
8. Can the authors provide an interactive online tool that allows users to explore the relationship among metabolites, RNA expression, metabolite pathway scores, gene pathway scores, and mutations? The figures in this manuscript are mostly high-level summary figures at pathway levels, I would like to see some compelling scatter plots and heatmaps that validate specific relationships. For example, it is hard for me to find out which of the metabolites in a pathway actually contributed most to the pathway score and correlation with orthogonal data, and whether the metabolites that fall into the same pathway really change concordantly.
Minor points:
1. Is this an original study? Under the method section, it states "Underlying data generated for this study is made available in Cherkaoui et al." But I cannot find the "Cherkaoui et al." reference.
2. Please provide Cellosaurus RRID for the cell lines in supplementary table 1 and KEGG ID in supplementary 2.
3. Please deposit codes used for the analyses.

Reviewer #3:

Shorthouse et al. presents a systematic metabolic characterization of 173 cancer cell lines measuring >1,000 metabolite measurements, representing one of the largest cancer cell line dataset currently generated. Harnessing the wealth of multi-omics datasets available for the same cell lines, the authors conducted a systematic discovery analysis of metabolic

associations with molecular (genomic and transcriptomic) and phenotypic (drug response) data to reveal novel metabolic associations with cancer genes and anticancer drug response. Particularly, they explore this approach to identify promising drug combinations and to reveal the distinct metabolic impact of specific mutations in cancer genes.

The authors make a very good use of the existing datasets for the same cell lines, by integrating with the generated metabolomics data to reveal interesting insights, for example, into the distinct metabolic phenotypes arising from specific mutations in the same cancer genes. Both these findings and the metabolomics dataset will be of great interest to the scientific community, for example, for preclinical studies where the availability of large-scale metabolomics is lagging behind compared to other omics. Other metabolomics datasets already exist - Li et al. (2019) quantified 225 metabolites across 928 cell lines - but Shorthouse et al. capitalizes on untargeted metabolomics to expand the metabolite measurements. Stronger integration with similar metabolomics data and some methodological improvements (e.g. more clarity and consistency on the confounding factors used in the association analyses) would strengthen the conclusions.

Major points:

1. Considering that an independent metabolomics dataset exists (Li et al. 2019) and that it is likely that cancer cell lines overlap, it would be important to assess the reproducibility of the metabolic measurements of cell lines screened in both studies. For example, can the authors correlate metabolites or pathway activities scores of the same cell lines and contrast this distribution with the random expectation (i.e. all-vs-all)?

2. Figure 2A and B are excellent examples of the power of this dataset to functionally annotate specific mutations. Have the authors seen any other associations with TCA cycle enzymes? Moreover, KRAS shows a predominant role in the metabolic associations. Given the frequent gain-of-function mutations, have the authors found any particular association specific to each mutation type?

3. For large-scale studies the heterogeneity across cancer cell lines is an important aspect that needs to be considered in order to avoid the detection of spurious associations. By fixing the same growing conditions for all cancer cell lines the authors have already taken an important step towards this aim. Are cancer cell lines growth rates (Li et al. 2019) related to any of the metabolomics PCs? Would there be any significant impact in the associations if controlled for growth (e.g. in drug-metabolite associations where growth rate is a strong determinant of drug response (Gonçalves et al. 2020))? Also in this context, in Page 5 second paragraph, it is difficult to understand how Figure S2 panels support the claim of the no large bias in the PCs of the metabolomics, particularly in light of the previous sentence, where cell volume is mentioned as a potential confounder. Could the authors elaborate on the link of cell size as potential confounder?

4. TFs activity profiles are very lineage dependent, was tissue type accounted for in the TF activity associations?

5. For the drug-metabolite associations, can these be corroborated using the single gene knockout CRISPR-Cas9 screens of the canonical drug targets (if known)?

6. The fact that hierarchical clustering did not provide any evident grouping by tissue is interesting, in line with previous metabolomics studies, and contrasting with other orthogonal cancer cell line molecular data (e.g. proteomics and transcriptomics). From Figure S2C some cell line clusters seem to exist, have the authors explored the potential major drivers of these groups?

7. The drug combination idea of pairing drugs with anticorrelated profiles of pathway associations is interesting. Are the proposed anti-correlated combinations recapitulated with recently experimental drug combinations in the same cells (Jaaks et al. 2022)?

Minor points:

1. Any particular technical reason to provide a T-map over some more traditional dimensional reduction clustering visualization methods, such as t-SNE or UMAP? Perhaps complete the current analysis with a two dimensional UMAP? How does the cell line grouping look before and after data normalization and correction?

2. The blood cancer cell lines were all grown in the same way, i.e. suspension vs attached?

3. Can the authors comment on the number of observations (cell lines) that are used to calculate the correlations at the tissue specific mutation analysis of Figure 5? Are these robust enough to calculate the associations?

4. For the NUTM2B and H3F3A associations with large effect sizes, could the authors provide the specific plot for, for example, the top association for each? It would be interesting to understand if this is driven by a single cell line/sample

5. Some of the plots labels are hard to read due to small font (e.g. Figure S1C, Figure 2E)

6. Could the authors provide more information about the statistical tests used in Figure 2E?

Reviewer #1:

In the present manuscript, Shorthouse et al investigate the landscape of metabolic profiles of 173 cancer cell lines grown under identical conditions. Using metabolomics data generated as part of an accompanying back-to-back manuscript (Cherkaoui et al) as their starting point, the authors integrate various types of orthogonal information to identify e.g. associations between metabolites and gene mutations, or between metabolic pathway activity and drug sensitivity.
Overall, this manuscript is well written and contains a plethora of interesting vignettes that will surely be of interest for the readership of MSB. However, there are currently several issues that should be clarified in a revised manuscript.

We thank the reviewer for their kind comments – we have done our best to address their concerns, making changes to the manuscript and text. Changes are described below, with page references. Changes to the text are highlighted in red.

Main comments:
1. Origin of metabolomics data: it is my understanding that the metabolomics data used by the authors is actually the same as the one generated by the accompanying manuscript from the Zamboni lab. However, this point is not actually spelled out in the text, and the methods section gives the somewhat misleading impression that this work also generated metabolomics measurements. I strongly suggest that the authors of both manuscripts clarify this point in their revised manuscript.

The reviewer is correct that both our manuscript and the accompanying manuscript from Cherkaoui et al is based on the same metabolomics data. We have clarified this in the text (lines 638-642).

2. Processing of metabolomics data: More importantly, it is unclear to me why this work used a quite different processing/normalization pipeline compared to the one from Cherkaoui et al, in which not only the number of ions considered, but also the number of cell lines are different. After doing some spot checks, I realized that the intensities for the same ions in the same samples seem to be quite different in the two studies (comparing suppl table 1 of this manuscript and the "injections" sheet of the Metabolomics data set provided as Primary analysis in the Zamboni lab manuscript), suggesting substantial differences in the output of the normalization pipelines. Given that the underlying data are the same (and taken from the Zamboni lab manuscript), I do not understand why the authors used a different normalization pipeline. If there is indeed a good reason for this decision, it is currently not communicated in the manuscript. I strongly suggest that the authors (of both manuscripts) either harmonize their data normalization pipelines or explain in detail why different ones are being used. Otherwise, I worry that having two conflicting data sets based on the same original data published back-to-back will lead to substantial confusion in the field.

The reviewer raises an important point. We would first note that we and Cherkaoui et al independently worked on this dataset, shared with us by our co-authors from AstraZeneca. The difference in normalisation procedure reflects this process, with our team focusing on an early normalisation procedure, whereas Cherkaoui explicitly sought alternative approaches. Only at a late stage of our individual research did we found that both groups had been analysing on the same data, and having found complementary results, subsequently sought to publish back-to-back.

We would further note that there does not exist a single, consensus normalisation approach across the field for this class of data. This is reflected in the analysis of alternative normalisation and analysis approaches presented in Cherkaoui. Whilst they sought to quantify "correctness", this in itself is dependent both on the specific measures that are used or excluded, and their relative

weighting used in the assessment. The choice of normalisation procedure may emphasise or obscure different features of the underlying data and therefore specific results may appear differently in the same dataset if differently normalised. In our work we were driven to analyse relationships between mutation and metabolite levels. As such we explicitly sought to functionally validate the data through the analysis of known relationships between mutants and metabolites, and having established this (figure 2) we continued to analyse other relationships in the data.

In light of these observations, we would hope that interpretation of analyses from either paper would be robust to the specific choice of normalisation procedure. We find that when repeating some of our analyses using the normalisation from Cherkaoui we reproduce our key findings, albeit with reduced effect sizes (Examples shown below). Notably, when examining the well-established relationships between mutations and metabolite we find that the impact is greatly reduced. This is inconsistent with known biology, and furthermore inconsistent with comparable published datasets, Li et al (https://doi.org/10.1038/s41591-019-0404-8), and Ortmayr et al (https://doi.org/10.1038/s41467-019-09695-9). A wider comparison of the normalisation procedures and published data additionally shows that multimodality is more common in the Cherkaoui dataset than either our dataset or other published work. Together, the sensitivity to mutations enabled by our approach and strong similarity to published datasets justifies our choice to retain our original normalisation scheme.

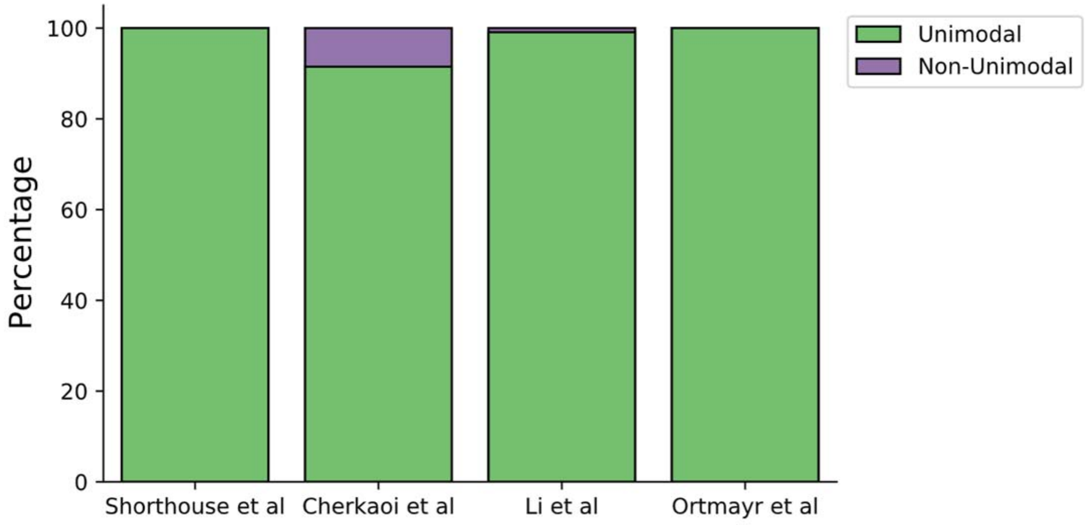

We however acknowledge the importance of clearly communicating the impact of the choices made in normalisation procedure, and to better support readers navigate the choice between procedures, we have provided an interactive dashboard that allows the exploration of the data generated from both this study and the co-submitted Cherkaoui et al. https://cancer-metabolomics.azurewebsites.net

Mutational Effects on Metabolite Accumulation

Shorthouse et al.

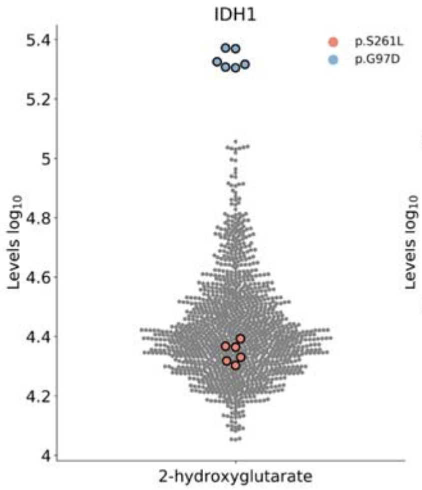

Cherkaoui et al.

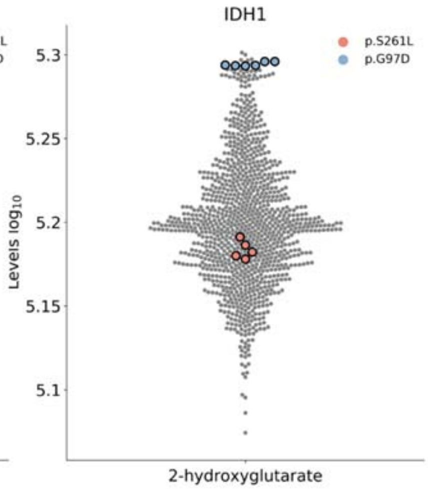

Pathway Activity Associations with Progeny Signatures

Shorthouse et al.

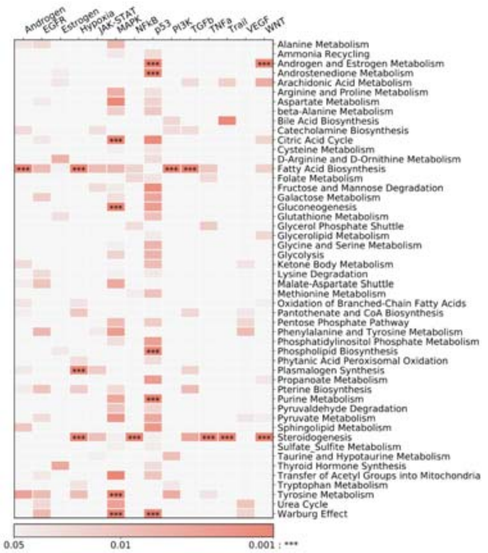

Cherkaoui et al.

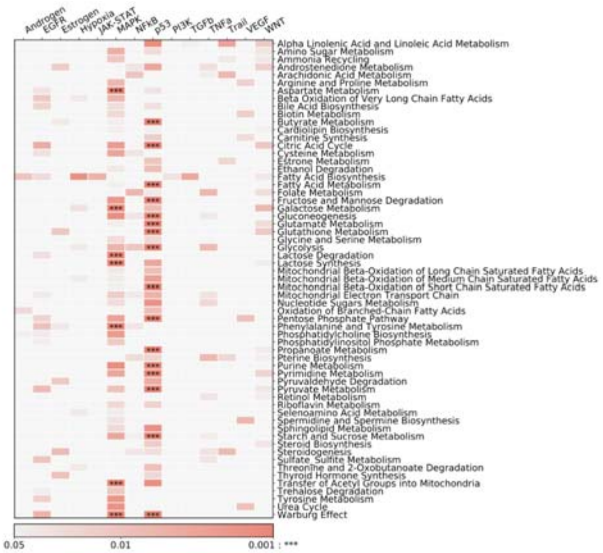

3. Relationship to previous literature: as the authors point out themselves, this is not the first attempt to comprehensively access the metabolic state of diverse cancer cell lines. For example, recent works have covered a larger cell panel, but with fewer metabolites measured (PMID 31068703), or a smaller cell panel (~60 cell lines), but with a similar number of measured metabolites (PMID 31015463). However, in the present manuscript the authors make little effort to put their findings in the broader context of the recent literature. For example, do any of the high-level conclusions of these recent studies hold up (or change) when expanding the metabolite/cell line dimension? I believe that this manuscript would benefit from an additional paragraph e.g. in the discussion tackling this point. Especially since it will allow the authors to articulate more clearly the "added value" this study provides to the field (which currently remains somewhat unclear).

We agree with the reviewer that a comparison with previous literature will add to this manuscript, and so have performed a comparison with two previously published datasets, Li et al (https://doi.org/10.1038/s41591-019-0404-8), and Ortmayr et al (https://doi.org/10.1038/s41467-019-09695-9). We have performed 3 comparisons:

- *We have confirmed that findings regarding mutation/metabolite accumulation are robust across datasets (Figure 2A).*
- *We have performed an analysis of the metabolite distributions across all three datasets, and show that the mean, standard deviation, and unimodality of the bulk metabolic measurements is consistent across all three studies. (Page 5, lines 125-132, Supplementary Figure 2)*
- *We compared metabolite levels for a number of matched metabolites across cell lines present both in our dataset and in Li et al, and find that when compared to a null distribution (generated by permuting the cell line order 1 million times) the correlations observed are statistically significant. This shows that the correlations between the datasets are significant. (Page 5, lines 125-132)*

We believe that these analyses highlight that our data reflects previously published data, and note that whilst Li et al covers a larger panel of cell lines, the number of metabolites measured is fewer than this study (225 vs 1099) making pathway analysis ability much narrower. We have added additional text discussing this and highlighting the added value of this study in the discussion. (Pages 26-27)

4. Figure 4B: I got confused regarding the exact metric being used here. In my understanding, the authors correlate (for a given drug) the IC50 value with a metabolic pathway activity metric across cell lines. A negative correlation between these two metrics would mean that higher pathway activity is associated with lower IC50 (and thus higher sensitivity to the respective drug). Since the log10 of the p-value is always going to be negative, I therefore expected positive products of log10(p-val) * Correlation_Direction to signify sensitivity. However, in figure 4B these are labeled as "resistant". Did the authors miss a (-) sign before the log10(p-val), or am I missing something?

We thank the reviewer for this comment – the axis label is intended to read "-log10(p-val)" in order to make it consistent with previous analysis, however the (-) sign was accidentally omitted. We have updated Figure 4B and the legend.

5. In general, I found Figures 4B-D very intriguing but hard to digest. For example, figure 4C is very interesting, but it didn't become clear to me a) why these particular two metabolic pathways were being highlighted here, and b) what the coloring scheme signifies. Similarly, in Figure 4D, I wasn't sure what type of correlation is being calculated here: is it the correlation of metabolic pathway sensitivities between different drugs? I suggest that authors add more detail on what exactly is being calculated in these plots.

We apologise for the lack of clarity here. To answer specific queries:

A) *We chose to highlight glycolysis and fatty acid biosynthesis as they are the two major pathways that show the highest anticorrelation with each other for drug sensitivity – ie. drugs that are resistant in cells that are high for glycolysis, tend to be sensitised in cells that are high for fatty acid biosynthesis.*
B) *We apologise for the omission here – we have added a legend to the figure to explain the colour scheme. Colours represent the difference between the relative resistance of each axis.*
C) *For figure 4D, we calculate the correlation of sensitivities for each drug against activity of each pathway, then for each pair of drugs we compare the sensitivities to generate a*

correlation between those two drugs – a positive correlation indicating that they are sensitive to the same metabolic pathways, and a negative correlation indicating that they are anticorrelated, and therefore may have a metabolic synergy. We have updated the text to explain this better (lines 394-395).

Additional comments:
1. Terminology: throughout the text, the authors refer to "upregulated" or "differently expressed" metabolites, and in one instance mention a mutation that is associated with a "loss of glutamine" (lines 249-250). I understand what the authors are trying to say, but I suggest that these terms (which make sense for gene expression, but not for metabolites) should be changed to e.g. "increased", "differently abundant". Similarly, I would suggest that the authors change the term "smaller doubling time" to the more conventional "shorter doubling time".

We agree with the reviewers suggestion, and have altered all instances of these terms as recommended.

2. Figure S1: in my understanding, the metabolomics method uses direct injection, without any chromatographic separation. To avoid confusion, I suggest that the authors remove the "LC-" prefix from panel A.

The reviewer is correct – we have altered the figure panel label (Figure S1A).

3. Line 140: I assume it's supposed to be Figure S2B (and not 2B), right? I also wasn't sure how to interpret this figure (and the accompanying lines 138-140): the argument is that all arrows in this plot have similar lengths, suggesting that no individual peak has a dominating effect on the PC's? An additional half-sentence may help clarify this point.

We thank the reviewer for finding this – the figure legend should indeed be Figure S2B (now S3B), and we accept that the figure could be better explained. Our argument is that the large number of principal components needed to explain the variation in the data, coupled with the lack of any strong associations of individual metabolites with PC1 and PC2 indicates that the data is not biased by external factors – such as cell volume, which was identified as a confounder through this method in a previous study: https://doi.org/10.1038/s41467-019-09695-9 . We have added an additional sentence to explain this (Lines 137-138)

4. Clustering shown in Figure 1A: The authors conclude that cell lines from the same tissue are generally clustering together (lines 147-148). I found this hard to see, especially since almost half of the cell lines are from the lung. Is there a way to provide a statistical measure of the "homogeneousness" of the different branches to back up this conclusion?

We have generated a statistical test of homogeneity based on the clustermap presented in supplementary figure 1C, as there is currently no easy way of statistically measuring the homogeneity of a complex branched topological shape. We took the order of tissues observed in the clustermap and randomly permuted them 100,000 times to generate a base distribution, and compared this to our observed clustering. We find that when the same cell lines are taken into account the clustering is significant ($p < 0.001$), when we average the same cell lines and compare tissue level clustering this significance is lost (p 0.4). Whilst there is an observable clustering of some cell lines by eye in the t-map, we cannot statistically validate this, and so have updated the statements made in the main text (Lines 140-142, 147-149), and included the statistics in the methods (Lines 708-717).

5. The authors' efforts to find associations between metabolites and gene mutations is very intriguing, in particular the associations between metabolic enzymes and their substrates/products. The IDH1 example the authors provide is illuminating (suggesting that only one of the two mutations found in the cell lines affects IDH1 catalytic activity). Have the authors considered to systematically look for such enzyme-substrate/product pairs to identify those mutations that may affect catalytic activity?

We have provided all the associations between missense mutations and metabolite levels in supplementary table 4. Additionally when generating figures for this manuscript we looked to known metabolic enzymes and their associated metabolites to validate our findings (IDH, FH, GLUT, SDH) and have presented the findings in the manuscript. There are unfortunately no correlations between many of these specific mutations and metabolites in our data aside from those presented, mainly because of a lack of damaging mutations in the cell lines studied. Looking at supplementary table 4 however, there is the potential for discovering new associations, such as Poly-ADP ribose, the most positively associated metabolite with CCND1 mutations, substrate of PARP enzymes and known to regulate the cell cycle: https://doi.org/10.1016/j.yexcr.2022.113163

To increase the accessibility of these interactions we have made the data available in an interactive dashboard where the user can choose metabolite/mutational pairings to study, as well as study pathways and drug sensitivity correlations. The dashboard is available at: https://cancer-metabolomics.azurewebsites.net

6. Figure 5B: the authors refer to NRAS and BRAF in the text, but I couldn't see the respective data in Figure 5B (only KRAS and HRAS).

We apologise for the confusion here – the text is incorrect, and we have adjusted it to correctly name KRAS and HRAS (Line 446).

Reviewer #2:

The paper from Shorthouse et al. presents a potentially incredibly useful data resource. They generated untargeted metabolomic profiling data entailing 1099 ions for 173 cancer cell lines. The authors performed extensive association analysis with mutation, transcriptomic, and drug sensitivity data, with most of the analyses performed at the pathway level. However, most of the analyses seem to be quite superficial, without in-depth functional validation.

We thank the reviewer for their comments – we have address their concerns below and in text, highlighted in blue.

Major points:
1. Throughout the paper, the author kept replicate data in their analyses. I highly recommend they collapse the data to cell line-level, i.e. pick only one representative "centroid" cell line and remove the other replicates.

Whilst some of our figures contain biological replicate data (eg. Figure 2A), we calculate our statistics and correlations using average (mean) values per cell-line. For example, all transcription factor, mutation, and drug sensitivity analysis is performed on cell-line averaged data. We have updated the methods text to better reflect this (Line 703-704). We have additionally replaced the swarmplots in figure 2 and supplementary figure 4 with cell line averaged figures.

2. Inter-replicate variation is much smaller than inter-cell strain variation. None of the statistical tests they performed has taken into account these different levels of variation.

Most of our analysis is performed on cell-line averaged (mean) values, and so these different levels of variation do not need to be taken into account. We have updated the methods to better reflect this, and have replaced the swarmplots in figure 2 with cell-line averaged equivalents. Additionally, we have further performed calculations on the statistical clustering of the cell lines accounting for, and not accounting for biological replicates. We find that cell lines are not significantly clustered by tissue when biological repeats are taken into account (p = 0.4), and have altered the text and methods to reflect this Lines 140-142, 147-149.

3. The data representation by including replicates might also be misleading. For example in 1A, the local clusters of replicates from the same cell line make it seem like cell lines of the same tissue origin cluster tighter than they really are.

We agree with the reviewer – we have updated the legend and text to better explain the figure, and also included supplemental analysis on clustering of cell lines to show that aside from blood and lung cell lines showing an apparent clustering, there is not a statistical significant clustering effect based on tissue origin as discussed previously.

4. In Figure 2, the authors tried to state that different mutations in IDH1 or SDHAF22 can result in different changes in the downstream metabolite. Such claims require validation by replacing WT IDH1 or SDHAF2 in the same reference cell line with the point mutations of interest to see if the metabolic changes from the parental cell line match with what they observed in Figures 2A and 2C. In the current analysis, the 6 dots for each mutation are really from 6 replicates of the same cell line. These different point mutations were compared in different cell line backgrounds, not isogenic backgrounds. They need to point out the cell lines for each of the different IDH1 or SDHAF2 mutations.

We note that we do not explicitly claim in the paper that the mutation/metabolite levels identified are causative, we instead state that metabolite levels are correlated with particular mutations, and that the protein structures support the mutation having a functional role, though we are aware that cell culture manipulation and analysis is required to explicitly prove the mutational effects. We have however updated the swarmplots to include only the average data, and have labelled the cell lines involved (Figure 2).

5. Pathway score was calculated and analyzed extensively in the paper. However, with shotgun metabolomics, the same ion could match many different metabolites. If these isobaric metabolites happen to fall into the same pathway, wouldn't the pathway score be inflated as an artifact? Can the authors show how would the results be affected if only one most abundant representative metabolite is picked for the ion?

We chose to use an iterative hypergeometric test to measure the significance of a pathway as this statistical measure is robust to outliers. Whilst we expect that some noise will be introduced into the system due to the overlapping assignments of some metabolites, we do not expect that this will significantly impact all pathways, as it is unlikely that all pathway metabolites will have multiple assignments that differ from the pathway, especially as many metabolites with similar molecular weights are in the same pathways.

6. I would like to see a comparison (distribution of metabolite-specific pairwise correlations across

shared cell lines for all the shared metabolites) with the existing CCLE metabolomics data to see the cross-study reproducibility of cell line metabolomics data.

In an effort to check that our data accurately matches with previously published work we have:

- *Confirmed that findings regarding mutation/metabolite accumulation are robust across datasets (Figure 2A) in terms of IDH mutations influencing accumulation of 2-hydroxyglutarate.*
- *Performed an analysis of the metabolite distributions across three datasets (Li et al, Ortmayr et al, this study), and show that the mean, standard deviation, and unimodality of the bulk metabolic measurements is consistent across all three studies. (Page 5, lines 125-132) supplementary figure 2.*
- *Compared metabolite levels for a number of matched metabolites across cell lines present both in our dataset and in Li et al, and find that when compared to a null distribution (generated by permuting the cell line order 1 million times) the correlations observed are statistically significant. This shows that the correlations between cell lines in the two datasets are significant. (Page 5, lines 125-132)*

7. Have p-values been adjusted for multiple comparisons for all of the related analyses (I only see it for RNA correlation)? If not, they should. Please indicate in the text accordingly.

P-values have been adjusted for multiple comparisons where appropriate. We have updated the text to better reflect this. (Lines 761, 769)

8. Can the authors provide an interactive online tool that allows users to explore the relationship among metabolites, RNA expression, metabolite pathway scores, gene pathway scores, and mutations? The figures in this manuscript are mostly high-level summary figures at pathway levels, I would like to see some compelling scatter plots and heatmaps that validate specific relationships. For example, it is hard for me to find out which of the metabolites in a pathway actually contributed most to the pathway score and correlation with orthogonal data, and whether the metabolites that fall into the same pathway really change concordantly.

Whilst the authors are not web developers, we developed and published an interactive dashboard that allows the exploration of the data presented in the manuscript. The tool allows the exploration of mutation/metabolite associations, transcription factor associations, and drug sensitivities. *https://cancer-metabolomics.azurewebsites.net*

Minor points:
1. Is this an original study? Under the method section, it states "Underlying data generated for this study is made available in Cherkaoui et al." But I cannot find the "Cherkaoui et al." reference.

This is an original study, however we and Cherkaoui et al independently worked on this dataset, shared with us by our co-authors from AstraZeneca. We have updated the manuscript text to better reflect this (lines 638-642).

2. Please provide Cellosaurus RRID for the cell lines in supplementary table 1 and KEGG ID in supplementary 2.

We have added the RRID and KEGG IDs to the relevant tables.

3. Please deposit codes used for the analyses.

We have uploaded all code used for analysis and generation of the dashboard at a github repository here (also accessible from the dashboard)): https://github.com/shorthouse-mrc/CellLine_Metabolomics

Reviewer #3:

Shorthouse et al. presents a systematic metabolic characterization of 173 cancer cell lines measuring >1,000 metabolite measurements, representing one of the largest cancer cell line dataset currently generated. Harnessing the wealth of multi-omics datasets available for the same cell lines, the authors conducted a systematic discovery analysis of metabolic associations with molecular (genomic and transcriptomic) and phenotypic (drug response) data to reveal novel metabolic associations with cancer genes and anticancer drug response. Particularly, they explore this approach to identify promising drug combinations and to reveal the distinct metabolic impact of specific mutations in cancer genes.

The authors make a very good use of the existing datasets for the same cell lines, by integrating with the generated metabolomics data to reveal interesting insights, for example, into the distinct metabolic phenotypes arising from specific mutations in the same cancer genes. Both these findings and the metabolomics dataset will be of great interest to the scientific community, for example, for preclinical studies where the availability of large-scale metabolomics is lagging behind compared to other omics. Other metabolomics datasets already exist - Li et al. (2019) quantified 225 metabolites across 928 cell lines - but Shorthouse et al. capitalizes on untargeted metabolomics to expand the metabolite measurements. Stronger integration with similar metabolomics data and some methodological improvements (e.g. more clarity and consistency on the confounding factors used in the association analyses) would strengthen the conclusions.

We thank the reviewer for their comments – we have responded to and addressed their comments below and in the manuscript, and corrections related to their suggestions are included in orange.

Major points:

1. Considering that an independent metabolomics dataset exists (Li et al. 2019) and that it is likely that cancer cell lines overlap, it would be important to assess the reproducibility of the metabolic measurements of cell lines screened in both studies. For example, can the authors correlate metabolites or pathway activities scores of the same cell lines and contrast this distribution with the random expectation (i.e. all-vs-all)?

In an effort to check that our data accurately matches with previously published work we have:

- *Confirmed that findings regarding mutation/metabolite accumulation are robust across datasets (Figure 2A) in terms of IDH mutations influencing accumulation of 2-hydroxyglutarate.*
- *Performed an analysis of the metabolite distributions across three datasets (Li et al, Ortmayr et al, this study), and show that the mean, standard deviation, and unimodality of the bulk metabolic measurements is consistent across all three studies. (Page 5, lines 125-132) supplementary figure 2.*

- *Compared metabolite levels for a number of matched metabolites across cell lines present both in our dataset and in Li et al, and find that when compared to a null distribution (generated by permuting the cell line order 1 million times) the correlations observed are statistically significant. This shows that the correlations between cell lines in the two datasets are significant. (Page 5, lines 125-132)*

2. Figure 2A and B are excellent examples of the power of this dataset to functionally annotate specific mutations. Have the authors seen any other associations with TCA cycle enzymes? Moreover, KRAS shows a predominant role in the metabolic associations. Given the frequent gain-of-function mutations, have the authors found any particular association specific to each mutation type?

For analysis of questions such as this we have developed a dashboard that allows the user to study relationships in the data of their choosing: https://cancer-metabolomics.azurewebsites.net

Moreover we have recalculated the regression mutation analysis for KRAS for each mutation type. We have added an additional paragraph to the text (line 245-247), as well as an additional supplementary figure and table to explain this data (Figure S8).

3. For large-scale studies the heterogeneity across cancer cell lines is an important aspect that needs to be considered in order to avoid the detection of spurious associations. By fixing the same growing conditions for all cancer cell lines the authors have already taken an important step towards this aim. Are cancer cell lines growth rates (Li et al. 2019) related to any of the metabolomics PCs? Would there be any significant impact in the associations if controlled for growth (e.g. in drug-metabolite associations where growth rate is a strong determinant of drug response (Gonçalves et al. 2020))? Also in this context, in Page 5 second paragraph, it is difficult to understand how Figure S2 panels support the claim of the no large bias in the PCs of the metabolomics, particularly in light of the previous sentence, where cell volume is mentioned as a potential confounder. Could the authors elaborate on the link of cell size as potential confounder?

We have identified a number of metabolic pathways and associated metabolites associated with cellular proliferation rate (Figure 1 D). Though we note that this analysis uses the reported doubling time from a different source (NCI-DTP) and is not collected by us. Our argument regarding PCA showing no strong biases is that the large number of principal components needed to explain the variation in the data, coupled with the lack of any strong associations of individual metabolites with PC1 and PC2 indicates that the data is not biased by external factors. That all the metabolites show a small and similar contribution to the PC's indicates that no single or small group of metabolites are driving variation in the data. This method previously identified that a group of metabolites were biasing the variation in a different study: https://doi.org/10.1038/s41467-019-09695-9 , and in this study the metabolites responsible for dominating the principal components were found to be associated with cell volume. We have added an additional sentence to explain this (Lines 137-138)

4. TFs activity profiles are very lineage dependent, was tissue type accounted for in the TF activity associations?

Whilst we do not explicitly take into account the tissue types when calculating TF associations, we instead calculate a p value for the correlation between transcription factor activity across all cell lines, and metabolite expression to see whether the rankings of metabolites across a particular pathway is higher than expected by chance. This method is robust to outliers and so the returned p-value (after multiple test correction) is a measure across all cell lines of whether metabolites in a

pathway are particularly highly or lowly accumulated in correlation with the studied TF, a single cell line that has high activity would not overly dominate this, as the method used (iterative hypergeometric test) is robust to single outliers dominating the variation.

5. For the drug-metabolite associations, can these be corroborated using the single gene knockout CRISPR-Cas9 screens of the canonical drug targets (if known)?

The reviewer raises an interesting point. Whilst this is theoretically possible, we have avoided attempting this analysis with public data because both the mechanisms of action of many drugs are unclear, and both the lack of clarity in the mechanism and off target effects which would confound an analysis, and necessitating further experimental work to assess. Such a study is however a natural extension to the work presented here, and we have made a note discussing the idea in the conclusions.

6. The fact that hierarchical clustering did not provide any evident grouping by tissue is interesting, in line with previous metabolomics studies, and contrasting with other orthogonal cancer cell line molecular data (e.g. proteomics and transcriptomics). From Figure S2C some cell line clusters seem to exist, have the authors explored the potential major drivers of these groups?

We have attempted to study these groupings to identify pathways/mutations that might correlate between them, but have been unable to identify strong differences between them – further work using more sophisticated methods such as machine learning tools may be able to better unpick differences, but as we were unable to find strong separation or large-scale differences in the data landscape, in this study we first validated that the data accurately reproduced known effects (such as pairings between mutations and metabolite accumulation/depletion) before performing more open-ended analysis.

7. The drug combination idea of pairing drugs with anticorrelated profiles of pathway associations is interesting. Are the proposed anti-correlated combinations recapitulated with recently experimental drug combinations in the same cells (Jaaks et al. 2022)?

Figure 4D represents an attempt to perform this comparison – the red boxes over the heatmap are combinations of drugs that are shown to be synergistic through an equivalent study to Jaaks et al. (https://doi.org/10.1093/nar/gkz1007) .

Minor points:

1. Any particular technical reason to provide a T-map over some more traditional dimensional reduction clustering visualization methods, such as t-SNE or UMAP? Perhaps complete the current analysis with a two dimensional UMAP? How does the cell line grouping look before and after data normalization and correction?

We originally attempted to cluster the data using UMAP, as well as TSNE, and other machine learning based clustering methods, but were unable to generate any real separation of the data due to its extreme homo/heterogeneity as noted in previous studies. We have attached an example of a TSNE, we note that the cluster on the left of the image is of MCF7 cell lines, which were injected in each batch to act as a control, but we found were overly biasing the data and so were removed before analysis.

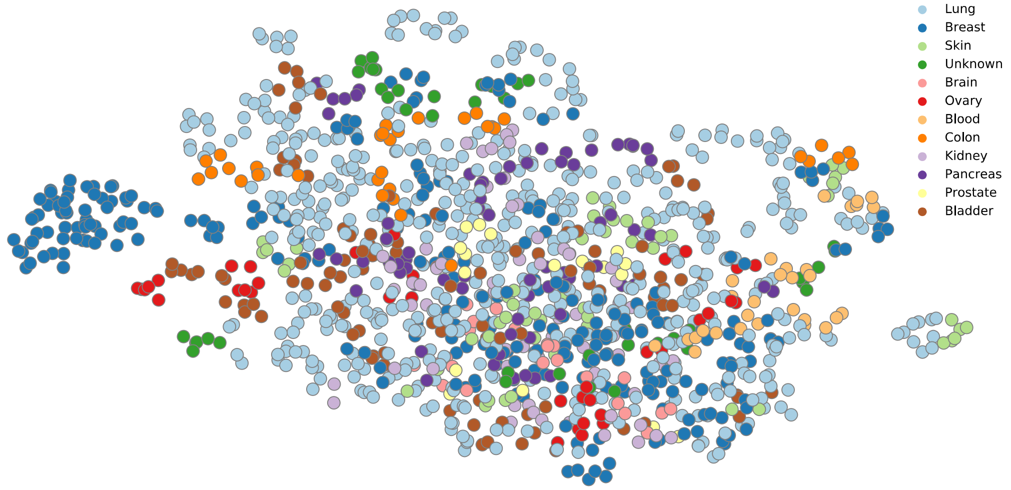

2. The blood cancer cell lines were all grown in the same way, i.e. suspension vs attached?

The blood cancer cell lines were all grown in suspension.

3. Can the authors comment on the number of observations (cell lines) that are used to calculate the correlations at the tissue specific mutation analysis of Figure 5? Are these robust enough to calculate the associations?

We only calculate these associations for the 50 most frequently mutated genes in order to ensure we are generating a landscape for genes where there is a number of mutations available. The gene with the lowest number of mutations included is found mutated in ~30 cells lines. For the most common cancer driver genes we find a larger number of cell lines, for example: TP53 113, KRAS 42, ATM 26. Whilst we cannot guarantee that some of the lesser mutated genes are robust as one tissue may only have one or two mutations, a large number of cell lines across all tissues are mutant for KRAS and TP53 for example.

4. For the NUTM2B and H3F3A associations with large effect sizes, could the authors provide the specific plot for, for example, the top association for each? It would be interesting to understand if this is driven by a single cell line/sample

These relationships can be explicitly studied using the dashboard we have generated as part of these revisions: https://cancer-metabolomics.azurewebsites.net

However the top associations for NUTM2B and H3F3A are as follows (taken from supplementary table 4):

NUTM2B

T-stat (rounded)	Peak	Metabolite Names
114	488	5'-Phosphoribosyl-N-formylglycinamide
52	492	Isorhamnetin

46	566	Topiramate/19-oic-deoxycorticosterone

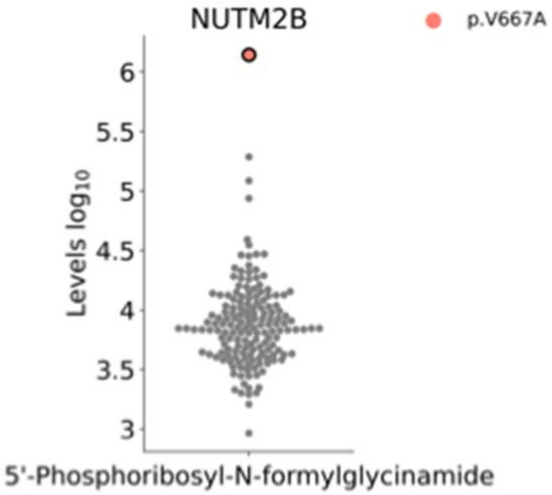

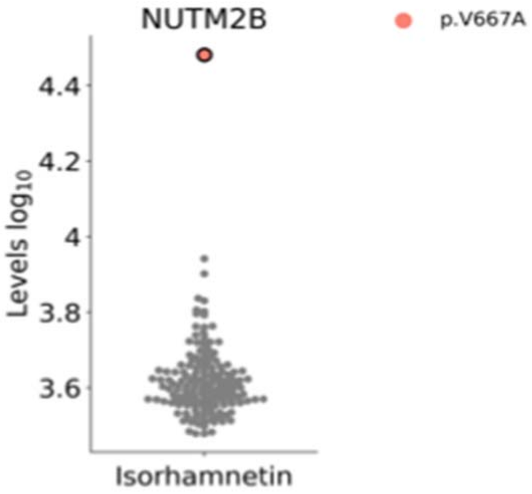

V667A mutation is found in SK-MEL-2.

H3F3A

T-stat (rounded)	Peak	Metabolite Names
116	617	3-Hydroxy-cis-5-tetradecenoylcarnitine
105	653	3-Hydroxyhexadecadienoylcarnitine
41	615	3-Hydroxy-5, 8-tetradecadiencarnitine
39	337	Isovalerylglutamic acid/Suberylglycine

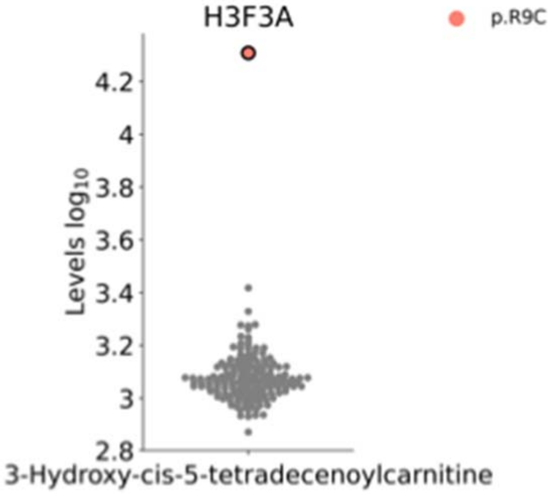

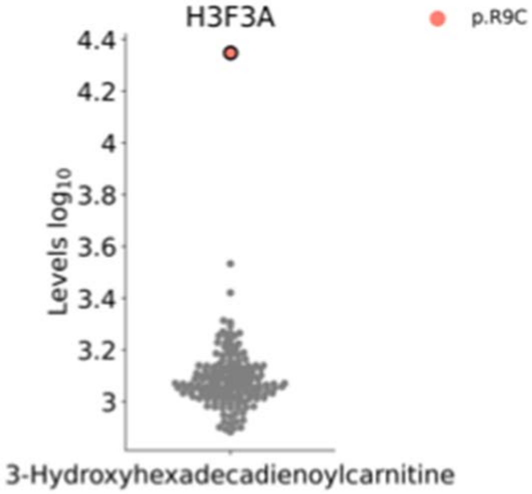

R9C mutation is found in NCI-H146.

5. Some of the plots labels are hard to ead due to small font (e.g. Figure S1C, Figure 2E)

We have improved the plot label font size where possible.

6. Could the authors provide more information about the statistical tests used in Figure 2E?

The enrichment is generated using the "enrichment analysis" tool included as part of the MetaboAnalyst package (also available at: https://www.metaboanalyst.ca/MetaboAnalyst/ModuleView.xhtml).

1st Revision - Editorial Decision **30th Aug 2022**

Thank you for sending us your revised manuscript. First, I would like to apologize for the delay in sending you a decision on your work. We have heard back from three reviewers who agreed to evaluate your study. As you will see, the reviewers are overall satisfied with the modifications made.

Before we can formally accept your manuscript, we would ask you to address the following issues:

1. The remaining minor issues raised by Reviewer #1.

On a more editorial level:

Reviewer #1:

I thank the authors for addressing all my concerns. I particularly appreciate the authors' efforts to make all data available in a dashboard. I am happy to recommend this manuscript for publication, and I only have a couple of minor comments:

1) Figure 1C still has the labels "upregulated/downregulated" when talking about metabolic pathway activity, I suggest that the authors use "increased/decreased" instead (as discussed in my original review)

2) In the PDF version I got, the legends of Figure 5D seem to be broken

Reviewer #2:

The authors have addressed my concerns. I especially applaud their efforts in creating a web application. It would be nice to upload the analysis code besides the web app code to the GitHub repo though. I am happy to recommend this manuscript for publication.

Reviewer #3:

The authors have satisfactorily addressed all my comments, the manuscript looks more supported with comparisons with other existing datasets. I have no doubt this will be an important study and resource for the community.

2nd Revision - Editorial Decision 7th Oct 2022

Thank you again for sending us your revised manuscript. We are now satisfied with the modifications made and I am pleased to inform you that your paper has been accepted for publication.

EMBO Press Author Checklist

Corresponding Author Name: Benjamin A Hall	
Journal Submitted to: Molecular Systems Biology	
Manuscript Number: MSB-2022-11006	

USEFUL LINKS FOR COMPLETING THIS FORM
The EMBO Journal - Author Guidelines
EMBO Reports - Author Guidelines
Molecular Systems Biology - Author Guidelines
EMBO Molecular Medicine - Author Guidelines

Reporting Checklist for Life Science Articles (updated January
This checklist is adapted from Materials Design Analysis Reporting (MDAR) Checklist for Authors. MDAR establishes a minimum set of requirements in transparent reporting in the life sciences (see Statement of Task: 10.31222/osf.io/9sm4x). Please follow the journal's guidelines in preparing your
Please note that a copy of this checklist will be published alongside your article.

Abridged guidelines for figures
1. Data
The data shown in figures should satisfy the following conditions:
- the data were obtained and processed according to the field's best practice and are presented to reflect the results of the experiments in an accurate and unbiased manner.
- ideally, figure panels should include only measurements that are directly comparable to each other and obtained with the same assay.
- plots include clearly labeled error bars for independent experiments and sample sizes. Unless justified, error bars should not be shown for technical
- if n<5, the individual data points from each experiment should be plotted. Any statistical test employed should be justified.
- Source Data should be included to report the data underlying figures according to the guidelines set out in the authorship guidelines on Data

2. Captions
Each figure caption should contain the following information, for each panel where they are relevant:
- a specification of the experimental system investigated (eg cell line, species name).
- the assay(s) and method(s) used to carry out the reported observations and measurements.
- an explicit mention of the biological and chemical entity(ies) that are being measured.
- an explicit mention of the biological and chemical entity(ies) that are altered/varied/perturbed in a controlled manner.
- the exact sample size (n) for each experimental group/condition, given as a number, not a range;
- a description of the sample collection allowing the reader to understand whether the samples represent technical or biological replicates (including how many animals, litters, cultures, etc.).
- a statement of how many times the experiment shown was independently replicated in the laboratory.
- definitions of statistical methods and measures:
  - common tests, such as t-test (please specify whether paired vs. unpaired), simple χ2 tests, Wilcoxon and Mann-Whitney tests, can be unambiguously identified by name only, but more complex techniques should be described in the methods section;
  - are tests one-sided or two-sided?
  - are there adjustments for multiple comparisons?
  - exact statistical test results, e.g., P values = x but not P values < x;
  - definition of 'center values' as median or average;
  - definition of error bars as s.d. or s.e.m.

Please complete ALL of the questions below.
Select "Not Applicable" only when the requested information is not relevant for your study.

Materials

Newly Created Materials	Information included in the manuscript?	In which section is the information available? (Reagents and Tools Table, Materials and Methods, Figures, Data Availability Section)
New materials and reagents need to be available; do any restrictions apply?	Not Applicable	

Antibodies	Information included in the manuscript?	In which section is the information available? (Reagents and Tools Table, Materials and Methods, Figures, Data Availability Section)
For **antibodies** provide the following information:<br>- Commercial antibodies: RRID (if possible) or supplier name, catalogue number and or/clone number<br>- Non-commercial: RRID or citation	Not Applicable	

DNA and RNA sequences	Information included in the manuscript?	In which section is the information available? (Reagents and Tools Table, Materials and Methods, Figures, Data Availability Section)
**Short novel DNA or RNA including primers, probes**: provide the sequences.	Not Applicable	

Cell materials	Information included in the manuscript?	In which section is the information available? (Reagents and Tools Table, Materials and Methods, Figures, Data Availability Section)
**Cell lines:** Provide species information, strain. Provide accession number in repository **OR** supplier name, catalog number, clone number, and/**OR** RRID.	Not Applicable	
**Primary cultures:** Provide species, strain, sex of origin, genetic modification status.	Not Applicable	
Report if the cell lines were recently **authenticated** (e.g., by STR profiling) and tested for mycoplasma contamination.	Not Applicable	

Experimental animals	Information included in the manuscript?	In which section is the information available? (Reagents and Tools Table, Materials and Methods, Figures, Data Availability Section)
**Laboratory animals or Model organisms:** Provide species, strain, sex, age, genetic modification status. Provide accession number in repository **OR** supplier name, catalog number, clone number, **OR** RRID.	Not Applicable	
**Animal observed in or captured from the field:** Provide species, sex, and age where possible**.**	Not Applicable	
Please detail **housing and husbandry conditions**.	Not Applicable	

Plants and microbes	Information included in the manuscript?	In which section is the information available? (Reagents and Tools Table, Materials and Methods, Figures, Data Availability Section)
**Plants:** provide species and strain, ecotype and cultivar where relevant, unique accession number if available, and source (including location for collected wild specimens).	Not Applicable	
**Microbes:** provide species and strain, unique accession number if available, and source.	Not Applicable	

Human research participants	Information included in the manuscript?	In which section is the information available? (Reagents and Tools Table, Materials and Methods, Figures, Data Availability Section)
If collected and within the bounds of privacy constraints report on age, sex and gender or ethnicity for all study participants.	Not Applicable	

Core facilities	Information included in the manuscript?	In which section is the information available? (Reagents and Tools Table, Materials and Methods, Figures, Data Availability Section)
If your work benefited from core facilities, was their service mentioned in the acknowledgments section?	Not Applicable	

Design

Study protocol	Information included in the manuscript?	In which section is the information available? (Reagents and Tools Table, Materials and Methods, Figures, Data Availability Section)
If study protocol has been **pre-registered, provide DOI in the manuscript**. For clinical trials, provide the trial registration number **OR** cite DOI.	Not Applicable	
Report the **clinical trial registration number** (at ClinicalTrials.gov or equivalent), where applicable.	Not Applicable	

Laboratory protocol	Information included in the manuscript?	In which section is the information available? (Reagents and Tools Table, Materials and Methods, Figures, Data Availability Section)
Provide DOI OR other citation details if **external detailed step-by-step protocols** are available.	Not Applicable	

Experimental study design and statistics	Information included in the manuscript?	In which section is the information available? (Reagents and Tools Table, Materials and Methods, Figures, Data Availability Section)
Include a statement about **sample size** estimate even if no statistical methods were used.	Yes	Materials and Methods
Were any steps taken to minimize the effects of subjective bias when allocating animals/samples to treatment (e.g. **randomization procedure**)? If yes, have they been described?	Not Applicable	
Include a statement about **blinding** even if no blinding was done.	Not Applicable	
Describe **inclusion/exclusion criteria** if samples or animals were excluded from the analysis. Were the criteria pre-established?<br><br>If sample or data points were omitted from analysis, report if this was due to attrition or intentional exclusion and provide justification.	Not Applicable	
For every figure, are **statistical tests** justified as appropriate? Do the data meet the assumptions of the tests (e.g., normal distribution)? Describe any methods used to assess it. Is there an estimate of variation within each group of data? Is the variance similar between the groups that are being statistically compared?	Yes	Materials and Methods, Supplementary Information

Sample definition and in-laboratory replication	Information included in the manuscript?	In which section is the information available? (Reagents and Tools Table, Materials and Methods, Figures, Data Availability Section)
In the figure legends: state number of times the experiment was **replicated** in laboratory.	Not Applicable	
In the figure legends: define whether data describe **technical or biological replicates**.	Yes	Materials and Methods

Ethics

Ethics	Information included in the manuscript?	In which section is the information available? (Reagents and Tools Table, Materials and Methods, Figures, Data Availability Section)
Studies involving **human participants**: State details of **authority granting ethics approval** (IRB or equivalent committee(s), provide reference number for approval.	Not Applicable	
Studies involving **human participants**: Include a statement confirming that **informed consent** was obtained from all subjects and that the experiments conformed to the principles set out in the WMA Declaration of Helsinki and the Department of Health and Human Services Belmont Report.	Not Applicable	
Studies involving **human participants:** For publication of **patient photos**, include a statement confirming that consent to publish was obtained.	Not Applicable	
Studies involving experimental **animals**: State details of **authority granting ethics approva**l (IRB or equivalent committee(s), provide reference number for approval. Include a statement of compliance with ethical regulations.	Not Applicable	
Studies involving **specimen and field samples:** State if relevant **permits** obtained, provide details of authority approving study; if none were required, explain why.	Not Applicable	

Dual Use Research of Concern (DURC)	Information included in the manuscript?	In which section is the information available? (Reagents and Tools Table, Materials and Methods, Figures, Data Availability Section)
Could your study fall under dual use research restrictions? Please check biosecurity documents and list of **select agents and toxins** (CDC): https://www.selectagents.gov/sat/list.htm	Not Applicable	
If you used a select agent, is the security level of the lab appropriate and reported in the manuscript?	Not Applicable	
If a study is subject to dual use research of concern regulations, is the name of the **authority granting approval and reference number** for the regulatory approval provided in the manuscript?	Not Applicable	

Reporting

The MDAR framework recommends adoption of discipline-specific guidelines, established and endorsed through community initiatives. Journals have their own policy about requiring specific guidelines and recommendations to complement MDAR.

Adherence to community standards	Information included in the manuscript?	In which section is the information available? (Reagents and Tools Table, Materials and Methods, Figures, Data Availability Section)
State if relevant guidelines or checklists (e.g., **ICMJE, MIBBI, ARRIVE, PRISMA**) have been followed or provided.	Not Applicable	
For **tumor marker prognostic studies,** we recommend that you follow the **REMARK** reporting guidelines (see link list at top right). See author guidelines, under 'Reporting Guidelines'. Please confirm you have followed these guidelines.	Not Applicable	
For **phase II and III randomized controlled trials**, please refer to the **CONSORT** flow diagram (see link list at top right) and submit the CONSORT checklist (see link list at top right) with your submission. See author guidelines, under 'Reporting Guidelines'. Please confirm you have submitted this list.	Not Applicable	

Data Availability

Data availability	Information included in the manuscript?	In which section is the information available? (Reagents and Tools Table, Materials and Methods, Figures, Data Availability Section)
Have **primary datasets** been deposited according to the journal's guidelines (see 'Data Deposition' section) and the respective accession numbers provided in the Data Availability Section?	Yes	Materials and Methods, Supplementary Information
Were **human clinical and genomic datasets** deposited in a public access-controlled repository in accordance to ethical obligations to the patients and to the applicable consent agreement?	Not Applicable	
Are **computational models** that are central and integral to a study available without restrictions in a machine-readable form? Were the relevant accession numbers or links provided?	Yes	Materials and Methods
If publicly available data were reused, provide the respective **data citations in the reference list**.	Not Applicable	
